# Harnessing the intragenomic variability of rRNA operons to improve differentiation of *Vibrio* species

**DOI:** 10.1038/s41598-024-60505-9

**Published:** 2024-04-30

**Authors:** Amaia Leunda-Esnaola, Evgeni Bunin, Pablo Arrufat, Peter B. Pearman, Vladimir R. Kaberdin

**Affiliations:** 1 Department of Immunology, Microbiology and Parasitology, University of the Basque Country UPV/EHU, 48940 Leioa, Spain; 2 Research Centre for Experimental Marine Biology and Biotechnology (Plentzia Marine Station, PiE-UPV/EHU), University of the Basque Country (UPV/EHU), Plentzia, Basque Country Spain; 3 CBET Research Group, Department of Zoology and Animal Cell Biology, University of the Basque Country (UPV/EHU), Leioa, Basque Country Spain; 4 Department of Plant Biology and Ecology, Faculty of Sciences and Technology, University of the Basque Country, UPV/EHU, Leioa, Spain; 5 IKERBASQUE, Basque Foundation for Science, Maria Diaz de Haro 3, 48013 Bilbao, Spain; 6 BC3 Basque Center for Climate Change, Scientific Campus of the University of the Basque Country, 48940 Leioa, Spain

**Keywords:** 16S and 23S ribosomal genes, Phylogenetic marker, Informative bases, Evolution, Bacteria, Classification and taxonomy

## Abstract

Although the 16S rRNA gene is frequently used as a phylogenetic marker in analysis of environmental DNA, this marker often fails to distinguish closely related species, including those in the genus *Vibrio*. Here, we investigate whether inclusion and analysis of 23S rRNA sequence can help overcome the intrinsic weaknesses of 16S rRNA analyses for the differentiation of *Vibrio* species. We construct a maximum likelihood 16S rRNA gene tree to assess the use of this gene to identify clades of *Vibrio* species. Within the 16S rRNA tree, we identify the putative informative bases responsible for polyphyly, and demonstrate the association of these positions with tree topology. We demonstrate that concatenation of 16S and 23S rRNA genes increases the number of informative nucleotide positions, thereby overcoming ambiguities in 16S rRNA-based phylogenetic reconstructions. Finally, we experimentally demonstrate that this approach considerably improves the differentiation and identification of *Vibrio* species in environmental samples.

## Introduction

The ubiquitous presence and diversity of microorganisms make them useful indicators of environmental changes that affect the integrity and functioning of aquatic ecosystems^1^. These systems currently experience substantial impacts in response to ongoing climate change caused by anthropogenic emission of greenhouse gases^2^. Among the bacteria used as environmental indicators in monitoring aquatic environments, species in the bacterial genus *Vibrio* are of particular interest due to their varied and important ecological roles and impacts^3,4^. Besides their involvement in nutrient cycling^5–7^, a number of *Vibrio* species are pathogens of humans (e.g., *V. cholerae, V. parahaemolyticus, V. vulnificus*)^8,9^ and of animals^10^. Vibrios can cause coral bleaching (*V. coralliilyticus, V. mediterranei*)^11^, bivalve mollusc mortality^12^, and a variety of lesions in crustaceans and fish^13,14^. Further, *Vibrio*-associated diseases are notable in the context of climate change, which has led to an increase in *Vibrio* outbreaks^15,16^ that have caused morbidity in humans^15^ and substantial economic losses in the seafood industry, especially in shrimp and fish aquaculture^14,17,18^. The increasing incidence of infection and losses involving *Vibrio*, the emergence of multi-drug resistant variants^13,14^, and economic and public health impacts, underscore the importance of rigorous environmental monitoring of these microorganisms.

As many marine and aquatic vibrios can be cultured in vitro, and a rough assessment of *Vibrio* community composition and dynamics can be carried out by using culture-based techniques (e.g., incubation on selective media such as TCBS agar or CHROMagar Vibrio). However, more complete information about community structure that includes both culturable and non-culturable (dormant) vibrios is obtainable with molecular tools such as CARD-FISH^19^ and analysis of metagenomic DNA extracted from environmental samples. Moreover, the culturable vibrios can subsequently be purified and genotyped to reveal species identities. In particular, comparative analysis of 16S rRNA gene sequences from the ribosomal operon, along with the use of other molecular tools (e.g., DNA-DNA hybridization; DDH and reverse transcription-polymerase chain reaction, RT-PCR) and phenotypic data, have promoted the accumulation of millions of ribosomal gene sequences in reference databases such as SILVA^20^ and rrnDB^21^. These archived sequences are currently used for species identification of vibrios and other bacteria^8,22^. Alternatively, the high genetic diversity and number of bacterial ‘housekeeping’ genes^23^ have made it possible to differentiate congeneric species, and to generate phylogenetic hypotheses based on comparative analysis, also known as Multilocus Sequence Analysis (MLSA)^24,25^. For some time MLSA has been used to differentiate *Vibrio* species and conduct taxonomic assignment of *Vibrio* isolates^26–28^. However, except for 16S rRNA, the use of house-keeping genes as phylogenetic markers is less common in analysis of more complex environmental DNA samples (eDNA), which contain the genetic material of multiple species. The complexity and multi-species composition of eDNA makes it difficult to ensure that chimeric genomes are not formed during sequence assembly, producing artificial genotypes. Further, the use of sequence from 16S rRNA alone results in incomplete species coverage during sequencing, as well as taxonomic assignment with limited accuracy^26,27^. Previous studies reveal that within-genome heterogeneity of ribosomal operons draws into question species discrimination based on the sequence of a single copy of the 16S rRNA gene^29,30^. For instance, 16S rRNA sequences from *Scytonema hyalinum* strains are extremely heterogenous as previously shown^31^, with an intra-genomic sequence variability of 7.3–9.0%, and their use frequently leads to incorrect taxonomic assignment. Similarly, the inconsistent differentiation of the genera *Butyrivibrio* and *Pseudobutyrivibrio*^32^ further demonstrates limitations of 16S RNA analysis. These findings highlight some intrinsic weaknesses of 16S rRNA as a phylogenetic marker. To overcome them, Martijn et al.^33^ employ the 16S and 23S rRNA genes to study bacterial and archaeal diversity in environmental samples and demonstrate higher statistical support and increased number of monophyletic groups in comparison to those obtained in phylogenetic analysis of variation at single genes. Nonetheless, the efficiency of this approach for species level differentiation in the *Vibrio* genus has not previously been studied. Likewise, an alternative approach using the ITS regions within 16S-23S rRNA has not provided sufficient resolution to unambiguously differentiate *Vibrio* species in earlier work^34,35^.

Here, we (i) define the key sequence features that limit the potential of 16S rRNA gene to serve as a phylogenetic marker in discrimination of *Vibrio* species and (ii) assess the capacity of 23S rRNA to improve species resolution in phylogenetic analysis. First, we reconstruct a phylogenetic tree by using 16S rRNA sequences retrieved from 40 completely sequenced *Vibrio* genomes. We evaluate whether nucleotide polymorphism within 16S rRNA *loci* of single *Vibrio* species genomes might cause polyphyly and taxonomic ambiguity. Further, we identify how variation at particular nucleotide positions in 16S rRNA gene can drive the formation of polyphyletic clades during phylogeny reconstruction, and demonstrate the role of these positions in determining tree topology. We show that concatenation of 16S and 23S rRNA genes increases the number of informative nucleotide positions, thereby overcoming ambiguities in 16S rRNA-based phylogenetic reconstructions and improving the differentiation of *Vibrio* species. We use these results to design and test *Vibrio*-specific PCR primers that target the conserved terminal regions of 16S and 23S rRNA genes in order to amplify complete 16S-23S regions. Our results will help to improve the detection and identification of *Vibrio* species in eDNA samples, thus facilitating *Vibrio* monitoring in aquatic ecosystems.

## Results

Through phylogenetic analysis of sequence variation among gene copies, we visualize the genetic variation within and among species that is represented in topological variation in phylogenetic trees. To minimize errors, our analysis was carried out with 40 *Vibrio* genomes selected based on (i) their completeness, and availability of high-quality sequencing data and annotation, along with (ii) preferentially unambiguous taxonomic assignment. The selected high-quality genomes belong to the groups that satisfy one of three levels of certainty. The first group include genomes that meet literature support and satisfy the NCBI taxonomic criteria check. The second group is limited to those that satisfy only NCBI taxonomic check. The third group contains those genomes that are not supported by any of the above criteria.

### 16S rRNA gene-based tree

**Figure 1:**
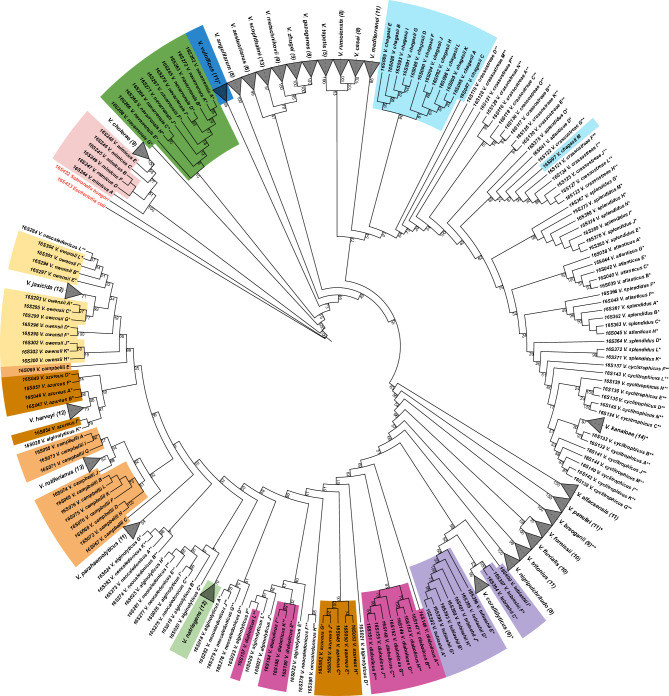
Maximum likelihood phylogenetic tree using all 16S rRNA sequences of 40 representative *Vibrio* genomes. Species are highlighted if they form clades in one of the phylogenetic trees (16S or 23S rRNA-based). Clades are collapsed and labelled with the corresponding species name. The number of gene copies in each clade is shown in brackets. The bootstrap values are calculated with 1000 replicates. The genome taxonomic assignment satisfies both reliability criteria (no asterisk), satisfies only the NCBI criterion (*) or does not satisfy any of the criteria (**). Two additional sequences corresponding to *Salmonella bongori* (16S422) and *Escherichia coli* (16S423) are indicated in red as an outgroup.

The topological analysis of 16S rRNA gene-based tree indicates that 16S rRNA gene copies of 26 species (e.g., *V. cholerae, V. vulnificus, V. casei*) form monophyletic clades (MCs), indicated on the tree by triangles (Fig. 1). Unlike a “cluster” that is usually referred to a group of sequences that bare resemblance to each other regardless of their evolutionary relationship, the phylogenetic term “clade” unites the group of sequences that belong to the organisms possessing a common ancestor. Our results demonstrate that 19 MCs are highly supported by bootstrap values ≥ 95 (Fig. 1), whereas other monophyletic clades, such as *V. parahaemolyticus* (bootstrap equal to 54) and *V. furnissii* (bootstrap equal to 70), are not sufficiently supported to fully rely on this monophyletic clade formation, based on variation at the 16S rRNA locus. The sequence variation present in the 16S rRNA copies of the remaining 14 species results in polyphyly, which is manifested by clustering of one or more 16S rRNA copies of one species with those of other species (e.g., *V. chagasii* in light blue and *V. azureus* in brown; Fig. 1). The species forming polyphyletic clades mostly coincide with the second and third level categories of certainty in genome taxonomic assignment that we defined (see Materials and Methods). Three distinct types of phylogenetic tree incongruences contribute to observed polyphyly: (i) ‘outlier’, the failure of one 16S rRNA copy to cluster with the rest of the copies from the same genome (e.g., *V. chagasii* in light blue*, V. campbellii* in orange, Fig. 1); (ii) ‘breaking’, a cluster of 16S RNA gene copies of one species is placed into what would otherwise be a MC of another species, resulting in paraphyly or polyphyly (e.g., *V. cholerae*—*V. mimicus* in light pink, *V. coralliilyticus*—*V. tubiashii* in purple, Fig. 1); (iii) ‘distinct clusters’, the appearance of two separate clusters of 16S rRNA gene sequences from the same genome (e.g., *V. azureus* in brown, Fig. 1).

### Disentangling polyphyletic patterns in the 16S rRNA gene-based tree

**Figure 2:**
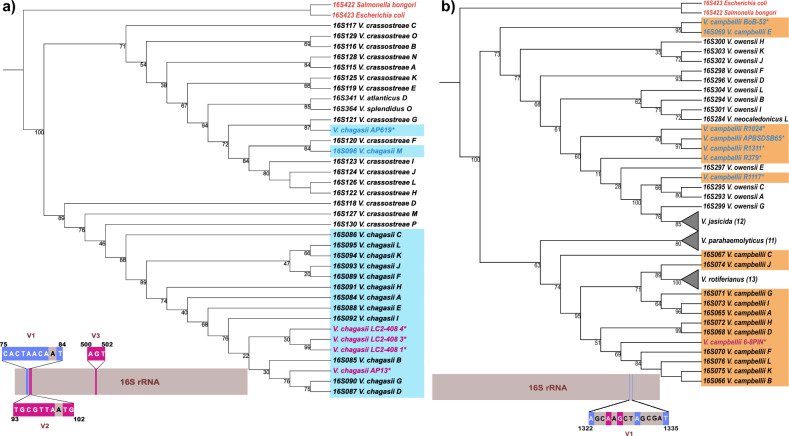
Maximum likelihood phylogenetic trees of *V. chagasii* (**a**, blue background) and *V. campbellii* (**b**, orange background) cases illustrating the outlier gene copies with a sister clade and the variable regions associated with outlier gene copy topology. A graphical representation of 16S rRNA gene with characteristic variable regions (V1, V2 and V3) is shown in the bottom left corner of each tree. The variable regions in blue are unique to the sequences (tree tips) featured with blue text, and variable regions highlighted with magenta are shared among sequences, indicated with both blue and magenta text. The black nucleotides inside of the variable regions are not unique to the outlier gene copy. Additional sequences with the highest homologies to the two species are from the SILVA database (*). Bootstrap support is calculated with 1000 replicates. The HKY + F + R2 substitution model is used here. Two additional sequences corresponding to *Salmonella bongori* (16S422) and *Escherichia coli* (16S423) are included as an outgroup (in red).

The phylogenetic ambiguity in the 16S rRNA tree appears largely associated with outlier gene copies and broken monophyly. The former is illustrated by patterns yielded by 16S rRNA copies in *V. chagasii* and *V. campbellii,* indicated by light blue and orange, respectively (Figs. 1 and 2). These gene copies present differing placements in the 16S rRNA tree, with one gene copy positioned separately from the rest of the clade, thus representing the case of an outlier gene copy (Fig. 1). The *V. chagasii* outlier gene copy M has three unique regions (i.e., V1, V2 and V3; Fig. 2a). Analysis of *V. chagasii* sequences from SILVA SSU data repository (indicated by a single asterisk in Fig. 2) reveals that they mainly contain variable regions 2 and 3, while only *V. chagasii* AP619 has all three variable regions (Fig. 2a).

In comparison, the outlier gene copy E of the *V. campbellii* genome has a variable region, V1, that is comprised of five unique nucleotides that are absent in other gene copies of the genome. However, the general composition of the V1 region is not unique. Further analysis shows that several *V. campbellii* sequences from the SILVA SSU data repository (Fig. 2b, indicated in blue) contain all five variable nucleotides. Moreover, one *V. campbellii* sequence (6-8PIN) has two variable nucleotides in this region (Fig. 2b, in magenta).

**Figure 3:**
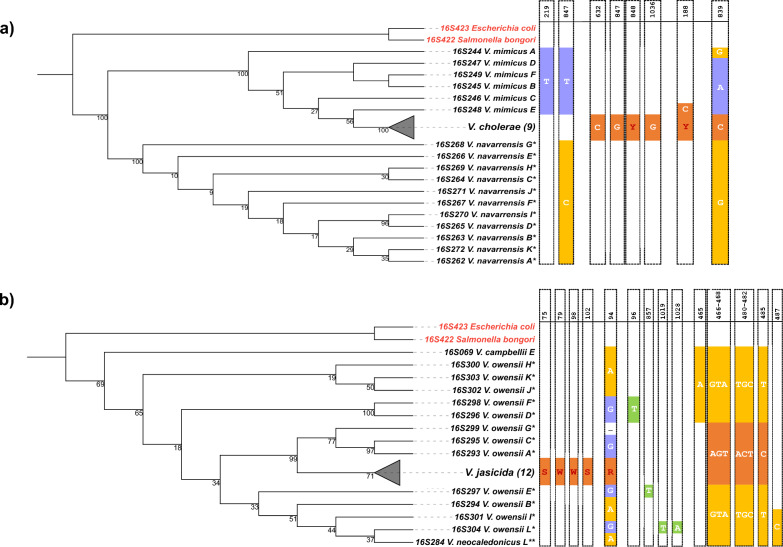
Maximum likelihood phylogenetic trees of *V. cholera*–*V. mimicus* (**a**) and *V. jasicida*–*V. owensii* (**b**), both cases forming a clade inside of a clade of another species. Informative nucleotides responsible for the species clustering patterns, i.e., nucleotides (in orange) of collapsed clades (i.e., *V. cholera* and *V. jasicida*) breaking another species clade; nucleotides (in blue) shared among gene copies of polyphyletic clades (*V. mimicus* and *V. owensii*); nucleotides (in yellow) belonging to sister species (*V. navarrensis*, *V. campbellii* and *V. neocaledonicus*); nucleotides (in green) associated with additional sequence sub-cluster, are specified. Sequences sharing the same nucleotide in the same position as one of the defined groups are indicated by the same color. Letter codes that represent degenerate nucleotides are in red (i.e., Y can be C or T; S can be G or C; W can be A or T; R can be A or G). The indicated nucleotides represent substitutions with one exception (i.e., deletion of one nucleotide in position 94 in gene copy “G” of *V. owensii,* indicated by hyphen), whereas the blank positions correspond to conserved nucleotides omitted for simplicity. The labelling by asterisks is the same as in Fig. 1. Bootstrap support is calculated with 1000 replicates. Two additional sequences corresponding to *Salmonella bongori* (16S422) and *Escherichia coli* (16S423) are included as an outgroup in red.

There are five instances of the second type of polyphyly, clade breaking, in the 16S rRNA tree (Fig. 1), in which a monophyletic clade is placed inside of another, otherwise monophyletic, clade. Examples include clustering of *V. cholera* within *V. mimicus* (light pink)*, V. jasicida* within *V. owensii* (yellow)*, V. harveyi* within *V. azureus* (brown), *V. rotiferianus* within *V. campbellii* (orange) and *V. coralliilyticus* inside of *V. tubiashii* (purple). To gain further insight, we present two cases (i.e., *V. cholerae* within *V. mimicus*, and *V. jasicida* within *V. owensii*) in further analysis. The incongruence in *V. mimicus* topology (Fig. 3a) is associated with several variable positions. Specifically, nucleotides in positions 219, 839, and 847 (highlighted in blue) are present in all gene copies of *V. mimicus*, while nucleotides in positions 632, 847, 848, and 1036 (highlighted in orange) are unique for *V. cholerae.* In addition, *V. mimicus* operon copy E exhibits cytosine (C) in position 188, a state shared with most 16S rRNA sequences of *V. cholerae*, while *V. mimicus* gene copy A has guanine (G) in position 839, which also occurs in all gene copies of *V. navarrensis*.

Several nucleotides are associated with the characteristic pattern of *V. owensii* 16S rRNA loci (Fig. 3b). Nucleotides in positions 75 (G), 79 (A), 94 (A/G), 98 (T), and 102 (G), highlighted in orange, are also present in most of *V. jasicida* sequences. Despite the presence of guanine (G) in position 94 in six *V. owensii* gene copies, other *V. owensii* gene copies (i.e., H, K, J, B and I) have adenine (A) in this location and therefore resemble sequences of *V. campbellii E* and *V. neocaledonicus L.* Additionally, *V. owensii* F, D, E and L have unique nucleotides in other positions (i.e., positions 96, 857, 1019 and 1028), highlighted in green. The remaining positions are either shared among *V. jasicida* and *V. owensii* gene copies G, C, and A (placed under a common node) or shared with other sister species, *V. campbellii* and *V. neocaledonicus.*

### 23S rRNA phylogenetic tree

**Figure 4:**
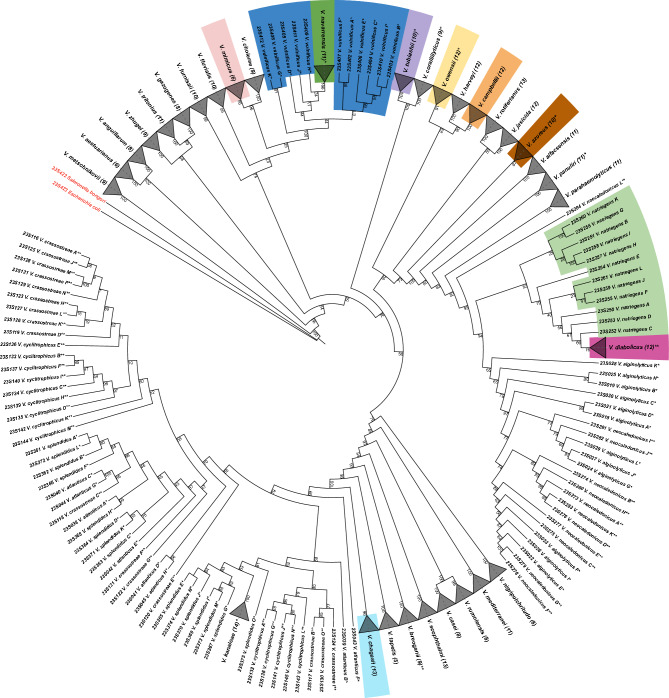
Maximum likelihood phylogenetic tree using all 23S rRNA sequences of 40 representative *Vibrio* genomes. Species are highlighted if they form clades in exactly one of the phylogenetic trees (16S or 23S rRNA-based). Clades are collapsed and the corresponding species names are indicated. The number of gene copies in each clade is shown in brackets. The bootstrap values are calculated with 1000 replicates. The labelling by asterisks is the same as in Fig. 1. Two additional sequences corresponding to *Salmonella bongori* (23S422) and *Escherichia coli* (23S423) are indicated in red as an outgroup.

Following the 16S rRNA tree-based strategy for 23S rRNA and further examination reveals that ribosomal sequences of 32 among 40 representative *Vibrio* species form MCs. *V. diabolicus* (magenta) and *V. owensii* (yellow) are the only species for which monophyly is not highly supported (bootstrap values equal to 76 and 91, respectively; Fig. 4). Polyphyly of an additional eight species is determined by one or several 23S rRNA gene copies clustering separately from the rest of the clade (e.g., *V. splendidus*, *V. crassostreae*, Fig. 4).

### 16S and 23S rRNA gene concatenation

**Figure 5:**
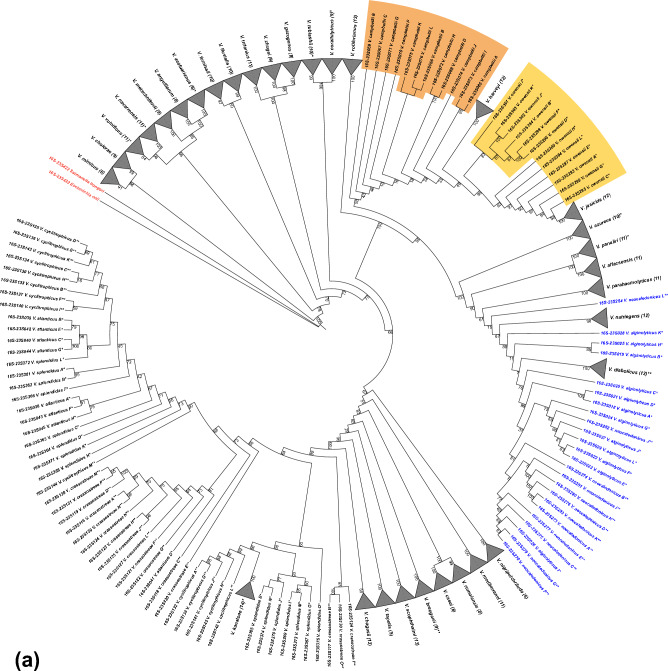

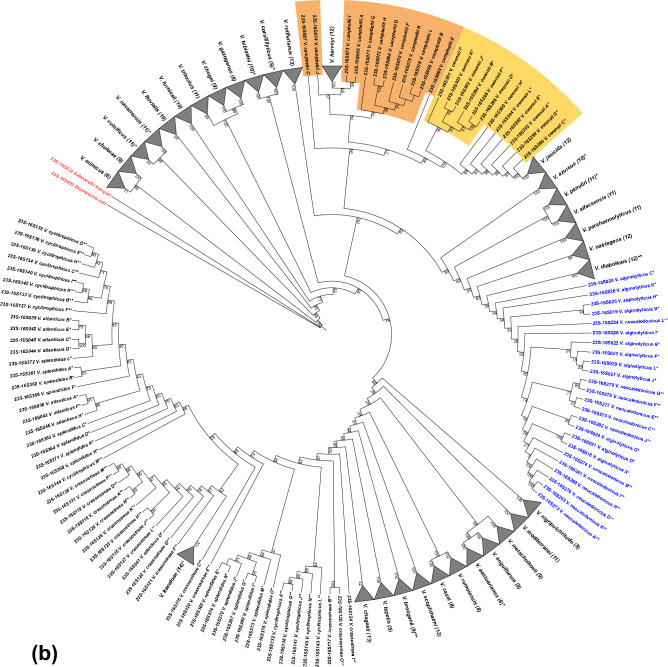
Maximum likelihood phylogenetic trees using 16S and 23S rRNA concatenated sequences in order 5′-16S-23S-3′ rRNA (panel **a**) or 5′-23S-16S-3′ rRNA (panel **b**) of 40 representative *Vibrio* genomes. To simplify the view, clades are collapsed and labelled with the corresponding species name. The number of gene copies in each clade is shown in brackets. Bootstrap support is calculated with 1000 replicates. *V. neocaledonicus* and *V. alginolyticus* are shown in blue. The labelling by asterisks is the same as in Fig. 1. Additional sequences corresponding to *Salmonella bongori* (16S-23S422 and 23S-16S422, respectively) and *Escherichia coli* (16S-23S423 and 23S-16S423, respectively) are indicated in red as an outgroup.

The above results indicate that some sequence features, including the lack of sufficient informative bases, can limit the number of MCs, especially when phylogenetic analysis is conducted with only one of either the 16S or the 23S rRNA genes. In an attempt to overcome this limitation, we alternatively generate phylogenetic trees by using concatenated 16S and 23S rRNA gene sequences (Fig. 5). Both types of 16S and 23S rRNA gene sequence concatenation (i.e., 16S-23S and 23S-16S) produce 32 MCs, of which the same 29 species are highly supported (bootstrap ≥ 95). We also show that the use of 16S-23S concatemers increases bootstrap support for clades of two species (*V. mimicus* and *V. chagasii*) and reduces support for *V. vulnificus*, (i.e., 91, 88 and 84, respectively; Fig. 5a) when compared to a 23S-16S tree (i.e., 71, 42 and 93, respectively; Fig. 5b). Moreover, although *V. neocaledonicus* and *V. alginolyticus* (Fig. 5b) form a monophyletic clade in the 23S-16S tree, this clade emerges as polyphyletic in the 16S-23S tree (Fig. 5a) due to insertion of *V. diabolicus* and *V. natriegens* sequences. In contrast to the MCs formed by *V. campbellii* (orange) and *V. owensii* (yellow) on the 23S rRNA tree (Fig. 4), these species do not form MCs in the 16S-23S and 23S-16S gene concatenation trees (Fig. 5a,b). However, the use of 16S-23S concatemers instead of 23S rRNA sequences better differentiates three species (*V. diabolicus, V. natriegens* and *V. vulnificus*; Figs. 4 and 5a) that were not resolved by 23S rRNA alone.

### Conserved and variable regions in Vibrio 16S and 23S rRNA genes

**Figure 6:**
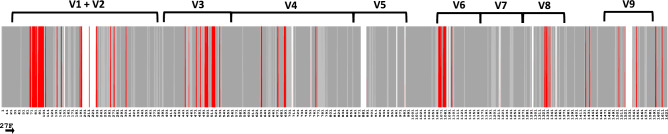
Variable and conserved regions of 16S rRNA and the location of 16S rRNA locus-specific forward primer. Sequence alignment of 16S rRNA gene sequences that are present in representative *Vibrio* genomes reveals the positions of variable (in red), fully conserved (dark gray), and conserved (in light gray) regions. The gaps are shown as white areas. Brackets indicate variable regions universally present in bacteria^30,36^. The position of 16S rRNA gene-specific forward (F) primer is shown at the bottom.

Since concatenation improves bootstrap support, we analyze further 16S and 23S rRNA loci to identify variable and conserved regions, and subsequently design *Vibrio*-specific primers for amplifying the entire 16S-23S genomic region. First, we find that the 16S rRNA locus is highly conserved across the *Vibrio* genus (Fig. 6). Approximately 74.6% of positions (1210) are fully conserved, whereas 162 positions are variable (Supplementary Table S1). Nine variable regions of 16S rRNA (indicated by brackets, Fig. 6) universally present in bacteria^30,36^ and variable regions identified in the alignment of *Vibrio* 16S rRNA sequences (indicated in red, Fig. 6) largely coincide. Regions 1, 3, 4, and 6 are the most variable in *Vibrio*, while regions 5, and 7 do not contain any variable positions except a 12-nucleotide-long insertion present in *V. metschnikovii* gene copy E, and guanine (G) insertion in *V. rumoiensis* gene copy B in variable regions 5 and 7, respectively (Fig. 6). Regions 2, 8, and 9 have some variable positions as well.

**Figure 7:**
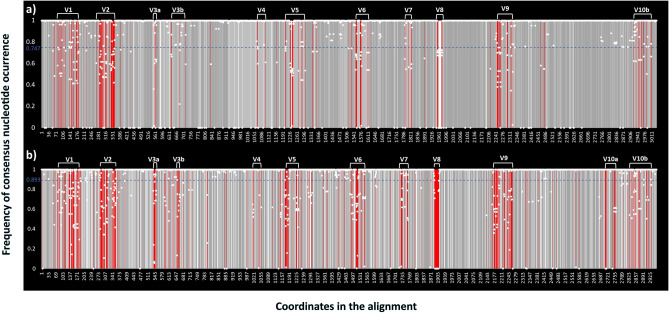
Deduced variable and conserved regions of 23S rRNA based on frequency values with which the consensus nucleotide at each aligned position occurs in *Vibrio* sequences retrieved from SILVA database (**a**) and a local data repository of representative *Vibrio* genomes (**b**). Indicated are fully conserved positions (dark gray), variable (red), conserved (light gray), and gaps (white). The position of the white dots representing individual consensus nucleotides is adjusted according to the frequency occurring at each position. A blue dashed line represents the cutoff frequency separating ten percent of the most variable nucleotides.

Second, our approximate delimitation of conserved and variable regions of *Vibrio* 23S rRNA sequences retrieved from SILVA LSU r138.1 RefNR data repository (Fig. 7a) reveals that 57.43% (1743) of the positions are fully conserved (Supplementary Table S1). By allowing a single mismatch in one of the aligned sequences per position, the conserved region can approximately cover 73.34% of the total alignment. In further interpretation of 303 variable positions (represented by red bars, Fig. 7a) we define variable regions as those with high concentration of variable nucleotide positions, i.e., those at which the consensus nucleotide occurs at frequencies lower than 0.747 (see Materials and Methods section and Fig. 7a). We find ten variable regions in aligned *Vibrio* 23S rRNA gene sequences. Moreover, variable region 3 is split into two subregions (i.e., 3a and 3b; Fig. 7a). In contrast to the alignment of 23S rRNA SILVA sequences, the 23S rRNA sequence alignment of our data repository (Fig. 7b) has a higher percentage of fully conserved regions (77.47%), which leads to a higher cutoff frequency value of 0.893 (Supplementary Table S1). The variable regions coincide in both alignments (Fig. 7), except for the presence of additional variable positions at the 3’ end (Fig. 7b). We include this additional region as V10a in Fig. 7b to differentiate shared variable positions in sequences retrieved from SILVA from those held in our repository.

### Selection of primers for amplification of Vibrio 16S-23S region

The assessment of several universal PCR primers allows selection of candidates for forward and reverse primers for the entire 16S-23S region (including both ribosomal RNA genes and intergenic spacer) in *Vibrio* genomes (Fig. 6, Supplementary Tables S2 and S3). Preliminary literature review reveals a main candidate location suitable for designing universal 16S rRNA gene-specific bacterial primers. This candidate location corresponds to primer variants similar to 27F, a universal forward primer widely used to amplify 16S rRNA bacterial gene sequences^37^. Furthermore, comparison of these variants provides a consensus primer sequence (c27F) representing all available primer variants (Supplementary Table S2). Although the universal bacterial forward primer (i.e., S-DBact-0008-cS-20, Supplementary Table S2) containing the same number of degenerate bases as c27F allows a better coverage among bacteria than a less degenerated primer, the *Vibrio*-specific consensus sequence eliminates the necessity of using such a highly degenerated forward primer for *Vibrio* species (Supplementary Table S2). We found that all custom *Vibrio* 16S rRNA gene copies can be amplified by the original 27F universal primer (5’-AGAGTTTGATCMTGGCTCAG-3’) introduced in 1991 (Supplementary Table S2).

After defining conserved regions in 23S rRNA genes (Fig. 7b), we consider two conserved regions as targets for a *Vibrio*-specific 23S rRNA reverse primer. Among them, the sense strand target of primer 23S_rev_V (positions 2864 to 2285) is closer to the 3’end of 23S rRNA sequence than the region in the sense strand (positions 2227 to 2243) complementary to primer 2242R. The first region provides a longer conserved sequence without indels and should provide nearly full length 23S rRNA amplicons, making 23S_rev_V the best candidate as a universal *Vibrio*-specific primer (Supplementary Table S3).

**Figure 8:**
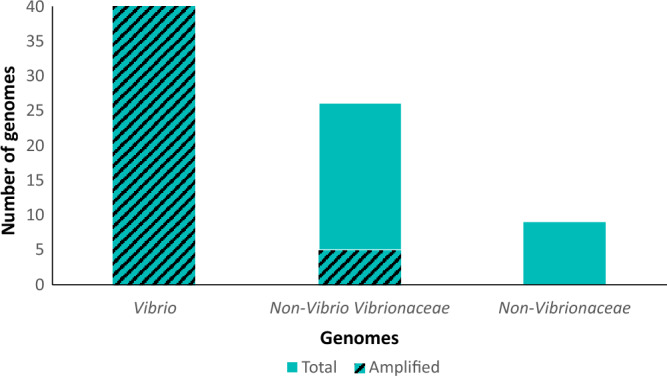
The results of in silico PCR amplifications obtained by the combination of 16S rRNA 27F^42^ and 23S rRNA 23S_rev_V primers. Whole genome sequences of *Vibrio*, non-*Vibrio* Vibrionaceae and non-Vibrionaceae species are used as templates. Genomes for which gene copies can be amplified (shaded) and can not be amplified (blue). The lack of amplification in the case of non-Vibrionaceae genomes suggests that the sequence of *Vibrio*-specific primers was not conserved in these genomes. Thus, the primers demonstrate preferential specificity for Vibrionaceae family.

An in silico specificity test employing the locus-specific 27F and 23S_rev_V primer pair for amplification of the 16S-23S region of complete *Vibrio* and non-*Vibrio* genomes does not yield products with non-Vibrionaceae genomes as templates (Fig. 8). In contrast, in silico PCR produces amplicons of anticipated length for all *Vibrio* genomes as well as for five non-*Vibrio* Vibrionaceae genomes (Fig. 8, Supplementary Table S4). Furthermore, the same primers amplify in silico all 16S-ITS-23S copies from 40 *Vibrio* genomes from our database (Fig. 8). All the amplicons obtained have the expected size, approximately 4700 bp.

### Experimental validation of *Vibrio*-specific primers

To experimentally test the ability of the *Vibrio*-specific primers to generate 16S-ITS-23S amplicons, we extracted metagenomic DNA from an environmental water sample and used it as a template. Consistent with the in silico results (see previous section), PCR amplification of environmental DNA using 27F and 23S_rev_V primers yields amplicons of the expected size (Supplementary Fig. S1). Their sequencing with Oxford Nanopore technology and standard protocols produced 105,230 reads (see “Materials and methods”; Supplementary Table S5). Analysis of high quality reads (83,207 in total) by WIMP (Oxford Nanopore) revealed that almost all reads belong to Proteobacteria (see Supplementary Fig. S2). Moreover, nearly half of these reads (48.9%) are of Gammaproteobacteria origin. We show that 2.17% reads correspond to the species that belong to the Vibrionaceae family (Supplementary Table S5). These reads (1,806 in total) represent a large variety of *Vibrio* species (see Supplementary Table S6). Interestingly, the trimming of 16S-ITS-23S reads at their 3’ end to obtain 1600 nt fragments (representing 16S rRNA gene sequences) and subsequent taxonomic annotation by WIMP (Oxford Nanopore) demonstrates that nearly one third of the truncated reads (i.e., 33.9%) become assigned to non-*Vibrio* species (Supplementary Fig. S2, panel *c*). Moreover, the trimming-dependent “loss” of some *Vibrio* reads also decreases the number of species compared to that initially discovered based on 16S-ITS-23S sequences (Supplementary Table S6).

## Discussion

Improved differentiation of *Vibrio* species has been possible using the 23S rRNA gene as a phylogenetic marker instead of the 16S rRNA gene. Analysis of a maximum likelihood (ML) 16S rRNA gene tree identifies the informative bases associated with multiple polyphyletic patterns (Figs. 2 and 3), which are largely resolved in a 23S rRNA gene tree. The 23S rRNA gene tree presents 11 additional highly supported monophyletic clades compared to the 16S rRNA gene tree (Figs. 1 and 4). The capacity of a 23S rRNA gene tree to reveal a higher number of monophyletic clades than a 16S rRNA gene phylogeny is consistent with the results obtained for non-*Vibrio* taxa^38^. The higher number of informative bases within the 23S rRNA gene when compared to 16S rRNA (i.e., 295 *vs* 162 variable positions; Supplementary Table S1) likely accounts for the observed increase in differentiation of *Vibrio* and non-*Vibrio* species.

We combine 16S rRNA and 23S rRNA gene sequences to increase the number of informative bases, in order to distinguish additional *Vibrio* species. The 16S-23S concatemer-based trees form a number of monophyletic clades similar to that provided by the 23S rRNA tree (Figs. 4 and 5, respectively), thus resembling the results for non-*Vibrio* species that are obtained with trees of single copies (per genome) of 23S rRNA and 16S-23S concatemers^38^. Nonetheless, the trees based on the concatemer sequences (Fig. 5) enable taxonomic assignment of three additional *Vibrio* species (*V. diabolicus*, *V. natriegens* and *V. vulnificus*). These species are not resolved in the phylogenetic trees based on *Vibrio* 23S rRNA or 16S rRNA sequences individually.

A new combination of 16S forward and 23S reverse primers for in silico amplification of *Vibrio* 16S-23S region suggests the feasibility of targeting *Vibrio*-specific sequences in environmental DNA*.* Amplification of this region in bacteria usually involves universal 16S rRNA 27F and 23S rRNA 2490R^39,40^ or 2241R^41^ primers. To increase the specificity of amplification, assure the broadest coverage of *Vibrio* species, and produce amplicons with an increased number of informative bases, we propose the combination of forward (16S rRNA 27F^42^) and new reverse (23S rRNA 23S_rev_V) primers. These primers can amplify in silico the corresponding fragments of all *Vibrio* ribosome operons in our custom database and provide products of 4.7 kbp or more. This size matches that of amplicons (i.e., 4.3–5.4 kbp) that encompass the entire 16S-ITS-23S regions in a large variety of bacterial species^41^.

Intragenomic variability in the number of rRNA operons constrains the use of ribosomal genes for analysis of environmental samples (https://rrndb.umms.med.umich.edu/search/)^21^. We observe that the number of ribosomal operons ranges from 5 to 16 for *Vibrio* genomes (Supplementary Fig. S3), and is on average higher than that reported in other studies^11,35^. Ribosomal operon multiplicity allows bacteria to increase ribosomal content quickly, providing rapid adaptation to changing environmental conditions, such as increase in nutrient availability or favorable temperature shifts^43^. Intragenomic variability in number of operons among strains that belong to the same species could lead to over- or underestimation of species richness, as the real number of species in samples can be lower/higher than that estimated based on the number of detected unique gene copies^30^. This circumstance restricts the application of ribosomal genes in quantitative analysis of eDNA^44^.

The general plasticity of bacterial genomes extends to the nucleotide composition of ribosomal operons, and therefore can influence outcomes of phylogenetic analysis. The presence of 16S rRNA and 23S rRNA in the same molecular machine (i.e. the ribosome) suggests their interdependent evolution to preserve ribosomal function. Compared to sequence conservation in regions important for ribosomal function, variable regions show higher diversity and are the location of informative bases in phylogenetic analysis. The phylogenies of our 16S rRNA and 23S rRNA gene copies from the same operon reveal different evolutionary relationships for some *Vibrio* species (Fig. 1 vs Fig. 4). These observations suggest that the routine sequencing of 16S rRNA amplicons and subsequent homology search can produce false matches for “outlier gene copies”, and consequently mislead species assignment. In this context, species assignment will benefit from fully annotated genomes based on all ribosomal operons^30,44,45^. The divergence of ribosomal operons can be explained by horizontal gene transfer or effects of mutation^46,47^. Even though gene transfer and mutations may increase intragenomic operon divergence in *Vibrio*, many studies report selection that favors homogeneous ribosomal structure and maintenance of function^47,48^. Intragenomic operon divergence may be transitory, and may provide an opportunity to study processes of operon homogenization.

Internal transcribed spacer (ITS; Supplementary Fig. S4) represents an additional source of informative bases contributing to the variability of the *Vibrio* ribosomal operon. Previous studies support the potential of ITS as a phylogenetic marker for differentiation of bacterial taxa from distinct families^39,41^. However, the effectiveness of ITS alone as a marker in a narrower range of taxa, such as species in the genus *Vibrio,* likely decreases. Consistent with this idea, the results of a previous study indicate that the use of ITS alone is insufficient for differentiating all *Vibrio* species^35^. Furthermore, lower intergenomic than intragenomic ITS sequence variability^33^ can further complicate species delineation based on ITS as a single marker. Despite some apparent limitations of ITS use, this region in combination with 16S and 23S rRNA genes could increase the total number of informative bases available for phylogeny construction and, therefore, might further improve the taxonomic assignment of *Vibrio* species.

We show through in silico analysis that, despite the key role of 16S rRNA gene in establishing the taxonomy of bacterial species, this gene possesses a number of deficiencies that complicate its use for differentiating *Vibrio* species in multispecies assemblages, such as those in environmental samples. Moreover, we demonstrate that some limitations can be overcome by the joint use of 16S and 23S rRNA genes, and we propose a candidate universal primer pair for *Vibrio*-specific amplification of the rRNA genes and the ITS. Although the joint use of ribosomal genes per se does not allow delineating all *Vibrio* species, the additional incorporation of ITS sequences present in the amplified 16S-ITS-23S fragments may increase the number of informative bases, potentially providing further improvements in the differentiation of *Vibrio* species in environmental samples.

The experimental testing of the proposed primers reveals that they work well with environmental DNA and are capable of amplifying a wide range of *Vibrio* sequences. Moreover, the use of these primers makes it possible to greatly increase the discoverability of *Vibrio* species compared to the “classical” 16S rRNA-based approaches widely used to monitor microbial diversity. For instance, one of the previous studies^49^ using 16S rRNA gene along as a phylogenetic marker apparently failed to identify any member of the Vibrionaceae family in environmental samples obtained from the same area (Plentzia Bay). Finally, 35.4% of *Vibrio* sequences that we discovered in the environmental sample originate from genomes that are not present among the genomes of 40 *Vibrio* species we initially selected for analysis. This strongly suggests that our primers enable broad coverage of *Vibrio* species.

## Materials and methods

### Creating a custom repository of 16S and 23S rRNA gene sequences

*Vibrio* is the most diverse genus in Vibrionaceae, currently including 151 described species and 5 subspecies (LPSN database, https://www.bacterio.net/, accessed June 2022)^50^. To carry out in silico analysis, we created a data repository by retrieving all copies of ribosomal operon genes (i.e., 16S rRNA and 23S rRNA) from 40 representative, fully-sequenced *Vibrio* genomes, one genome per species (Supplementary Table S7). Genome taxonomic assignment was further verified when *Vibrio* spp. didn’t form highly supported and unambiguously differentiated monophyletic clades. We classified levels of certainty of genome taxonomic assignment in the following way: first, literature support existed and the NCBI taxonomic check criteria were satisfied; second, only the NCBI taxonomic check criteria were satisfied; and third, when none of these criteria were satisfied (Fig. 1, Supplementary Table S8). When multiple genomes were available, we preferentially selected published and annotated genomes of validated *Vibrio* species in the LPSN database that were assembled using both long- and short-read sequences (e.g., those obtained by both PacBio and Illumina sequencing). To choose representative genomes of *V. diabolicus*, *V. natriegens,* and *V. scophthalmi* from IMG/M database (https://img.jgi.doe.gov/cgi-bin/m/main.cgi), we constructed a similarity matrix of gene copies from the same genome based on NCBI BLASTn results (https://blast.ncbi.nlm.nih.gov/) and analyzed the number of gaps and mismatches to find the genomes with the highest internal variability in 16S and 23S rRNA gene copies. Next, the ribosomal sequences that were downloaded from NCBI GenBank and IMG/M databases (Supplementary Fig. S5) were manually curated by adding missing conserved terminal nucleotides to obtain full-length copies. We assigned to each retrieved sequence a unique ID in which the last three digits referred to the operon carrying the corresponding 16S and 23S rRNA gene copies and a letter to distinguish each operon within the corresponding genome. We employed our custom code (Parts 1–5, see supplementary file “Custom code”) based on the automated webpage scraping functionality in the *RSelenium* (Version 1.7.7)^51^ and *rEntrez* packages (Version 1.2.2)^52^ to formulate a search query in R (Version 1.1.442) to obtain species and strain names, sequence accession numbers, and the corresponding sequences in FASTA format.

We obtained additional 16S rRNA sequences from the SILVA SSU r.138.1 database^20^. We used these sequences to ascertain whether outlier gene copies were fortuitous and potentially caused by sequencing errors, or occur more broadly in a larger sample of sequenced genes. We conducted a BLASTn homology search with the variable regions of outlier gene copies *V. chagasii* M and *V. campbellii* E. We subsequently used the five SILVA sequences with complete 16S rRNA sequence and the highest BLAST homology in polyphyly analysis of the 16S rRNA-based phylogenetic tree (Fig. 2). Additionally, 2072 non-redundant *Vibrio* 23S rRNA sequences were also retrieved from SILVA LSU Ref NR r.138.1 database^20^, corresponding to 45 species and 19 additional strains without species designation. These were then used to locate 23S rRNA conserved regions for PCR primer design (Fig. 7a, Supplementary Fig. S5). We further supplemented our repository with 26 genomes that belong to non-*Vibrio* species in Vibrionaceae and nine other non-Vibrionaceae bacteria. The non-*Vibrio* Vibrionaceae genera included *Aliivibrio*, *Photobacterium*, *Salinivibrio*, *Enterovibrio* and *Grimontia*, whereas non-Vibrionaceae families included Woeseiaceae, Comamonadaceae, Rhodobacteraceae, Desulfobacteraceae and Enterobacteriaceae (*Escherichia coli*).

### Alignment, curation, trimming and concatenation of rRNA gene sequences

We used MAFFT (Version 7.490) with a global strategy (G-INS-I)^53^ for sequence alignment. The MAFFT algorithm provided better-aligned sequences than those obtained by using other popular algorithms such as MUSCLE and ClustalW, based on inspection. After alignment, several incomplete and apparently misannotated 16S and 23S rRNA sequences were identified and subsequently curated using the full genome sequences previously retrieved from NCBI GenBank in order to assure a standard, full-length representation of gene sequences (Supplementary Fig. S5). Moreover, some aligned sequences were manually trimmed in MEGA-X at their 5’- and 3’ extremities to ensure uniform length of sequences flanking the conserved rRNA regions across all aligned sequences.

### Phylogenetic reconstruction

We used MAFFT to align and MEGA-X^54^ to curate rRNA gene sequences and the fusion variants (i.e., 5’-16S-23S-3’ and 5’-23S-16S-3’) obtained by concatenation of 16S and 23S rRNA from the same operon. These were then used to construct phylogenetic trees. Tree construction was performed in IQTREE (Version 2.1.3)^55^ at operon resolution using ML. The IQTREE algorithm automatically chose the best nucleotide substitution model for each case by selecting the model with the lowest Bayesian Information Criterion value (i.e., TIM3 + F + R5 for 16S rRNA, GTR + F + R7 for 23S rRNA, GTR + F + R7 for 16S-23S, and GTR + F + R6 for 23S-16S rRNA concatemers, respectively). IQTREE employs bootstrapping to describe node support in the reconstructed trees. We considered a node highly supported when the bootstrap value was ≥ 95^56^. We visualized the NEWICK format output file (.*treefile*) using the online tree editing program iTOL (Version 6, https://itol.embl.de/upload.cgi)^57^. We edited trees for better presentation using the vector graphics program Inkscape (Version 1.0, https://inkscape.org/release/inkscape-1.0/).

To interpret ambiguous sequence topologies in the 16S rRNA tree of the *V. chagasii* gene copy M and *V. campbellii* gene copy E (outlier gene copies), we visually identified the regions that are associated with differences between these outlier gene copies and other gene copies from the same genome. To assure that the unique sequence composition defining these regions was not artifactual (e.g., sequencing error), we checked for nucleotide conservation among other sequences of the same species by performing BLASTn search (https://blast.ncbi.nlm.nih.gov/) of these unique regions in additional *V. chagasii* and *V. campbellii* sequences from the SILVA SSU r.138.1 database^20^. Further, we employed Gblocks software^58^ to compare aligned 16S rRNA sequences of *Vibrio* spp. that form monophyletic clades that disrupt otherwise monophyletic *Vibrio* spp. clades (e.g., *V. cholera–V. mimicus, V. jasicida–V. owensii, V. harveyi–V. azureus, V. rotiferianus–V.campbellii,* and *V. coralliilyticus–V. tubiashii*). We refer to these cases as “broken” monophyly. Among the variable positions highlighted by Gblocks, we identified by inspection the informative nucleotides (i.e., nucleotide variants in some positions of 16S rRNA that were shared with closely related *Vibrio* species) that were associated with a particular topology in the phylogenetic tree. The IQ-TREE output file of the full 16S rRNA phylogenetic tree was pruned to point to a node with a distinct topology with regard to its polyphyletic sister clade and an outgroup.

### Variable region identification

To identify variable regions, we first used *Biostrings* R package (Version 2.64.0)^59^ employing our custom code (Part 6, see supplementary file “Custom code”) to score the sequence conservation for each nucleotide present in the curated and trimmed alignment of rRNA gene sequences. The consensus sequence was determined by the highest frequency base at each nucleotide position. Later, positions were sorted by these values. We considered variable positions to be the ten percent of positions with the lowest frequency values, x (i.e., x < 0.768 for 16S rRNA, x < 0.893 for representative *Vibrio* 23S rRNA and x < 0.747 for SILVA 23S rRNA sequences), while the remaining positions were referred to as partly conserved (0.768 ≤ x < 1, 0.893 ≤ x < 1, and 0.747 ≤ x < 1 for 16S rRNA and 23S rRNA representative *Vibrio* and SILVA sequences, respectively) or highly conserved (x = 1). Finally, we visually identified variable regions as containing a relatively high frequency of variable positions. The location of the variable regions was further corrected and refined based on comparison with the 16S variable regions reported in other studies, as well as through analysis of a larger set of *Vibrio* sequences available in SILVA LSU Ref NR database for 23S rRNA.

### Design of primers for amplification of the full-length 16S-23S regions of Vibrio genomes and their in silico testing

We assessed the degree to which 10 forward 16S rRNA gene-specific primers (Supplementary Table S2), previously used to amplify 16S rRNA genes, successfully hybridize with *Vibrio* 16S rRNA gene sequences. Among the primers that hybridize *Vibrio* 16S rRNA sequences, 27F primer, with a single degenerate base, provided sufficient coverage of *Vibrio* sequences.

To design 23S rRNA gene-specific primers, the longest conserved regions at the 3’ termini of aligned 23S rRNA sequences of representative *Vibrio* species were chosen as potential targets for new primers (Supplementary Table S3). To ensure base pairing in the variable positions, we allowed for primers with degenerate bases (e.g., Y represents C or T; R represents A or G; H represents A, C or T). We also included two universal 23S rRNA bacterial primers that target an internal region of this gene to assess the ability of these primers to amplify *Vibrio* sequences in our data repository. The location of primer binding was assessed using BLAST and the *Vibrio* sequences in the SILVA LSU r138.1 Ref NR database.

We tested the suitability of two primers, the 16S rRNA gene-specific 27F (5’-AGAGTTTGATCMTGGCTCAG-3’) and 23S rRNA gene-specific 23S_rev_V (5’-TARRHCTCAYGGGYRATTAGTR- 3’), to serve as universal primers for amplification of nearly full-length 16S-23S region, using the in silico PCR Experiment Simulation System (Ipcress; 2.2.0 exonerate, glib version 2.47.0). The amplification targeted sequences in our custom repository of *Vibrio*, non-*Vibrio* and non-*Vibrio* Vibrionaceae genomes, and we specified conditions that allowed up to three nucleotide mismatches and set up the upper limit for the length of amplicons (i.e., 6000 nt). 

### Water sample processing and extraction of metagenomic DNA

The environmental water sample (ES_Ple_Mar) was collected in the Estuary of Plentzia in March of 2023. One and a half liter of water collected from the surface and prefiltered through a 200 μm mesh was sequentially filtered through a 3 μm polycarbonate filters (142mm diameter) followed by a 0.22 μm Sterivex™ filter unit (Millipore) using a MasterFlex Easy-Load peristaltic pump. The sterivex filter with the attached biological material was further used to extract metagenomic DNA following the DNeasy PowerWater Sterivex Kit (Qiagen) protocol. The concentration of the extracted DNA (25.8 ng/μL) was determined using a Qubit 4 Fluorometer (Thermo Fisher Scientific).

### PCR amplification and gel purification of 16S-ITS-23S amplicons

The standard mixtures (50 μL) used to carry out PCR contained 19 μL of molecular biology grade water, 2.5 μL of forward primer (20 pmol/μL), 2.5 μL of reverse primer (20 pmol/μL), 1 μL of template DNA and 25 μL of 2 × Platinum SuperFi II Green PCR Master Mix (Thermo Fisher Scientific). PCR was performed by using a Veriti Thermal Cycler (Applied Biosystems, USA). The amplification process included an initial denaturation step (30 s, 98 °C) and 35 cycles of amplification (denaturation for 10 s at 98 °C, annealing for 10 s at 60 °C and extension for 2.5 min at 72 °C) followed by a final incubation for 5 min at 72 °C. The products of PCR amplification contained in two PCR tubes (100 μL in total) were deproteinized by extraction with an equal volume of phenol: chloroform: isoamyl alcohol (25:24:1). Then, an aliquote of the deproteinazed sample (6 μL) along with Gene Ruler 1kb Plus DNA Ladder (Fisher Scientific, USA) were further analysed by electrophoresis in an 1% agarose gel, followed by staining with GelRed (Millipore) and destaining in distilled water. The image of the destained gel was captured by using a ChemiDoc imaging system (Bio-Rad). To increase the yield of the target PCR product, it was reamplified using the deproteinized amplicon as a template. Aliquotes of the amplified products (50 μL each) were individually mixed with 10 μL of ROTI®Load DNAstain 2 SYBR® Green (Carl Roth) and were subsequently fractionated on a 1% agarose gel in triplicate. The DNA fragments of appr. 4500–5000 bp were visualized using a Large Blue LED Transilluminator (IO Rodeo) and extracted from the gel using the GeneJET Gel Extraction Kit (Thermo Scientific). The concentration of the extracted DNA (69.52 ng/μL) was determined using a Qubit 4 Fluorometer (Thermo Fisher Scientific).

### Oxford Nanopore sequencing and post-sequencing data processing

Purified 16S-ITS-23S amplicons were further used for preparation of library conducted following the Native Barcoding Kit 24 V14 protocol instructions (SQK-NBD114-24. Oxford Nanopore Technologies, ONT, Oxford, UK). The resulting library was loaded to the MinION Mk1B flow cell FLO-MIN114 for sequencing. The obtained reads were basecalled by Dorado basecaller, installed in MinKNOW, employing the High-accuracy basecalling model, 400bps-5 kHz chemistry and the default minimum quality score (Qscore = 9). All 16S-ITS-23S reads above Qscore nine were automatically grouped by MinKNOW in fastq files. The taxonomic classification and quantitative analysis of these reads was performed by EPI2ME Desktop Agent version 3.7.3 using “What’s in my pot” workflow (WIMP 2023.06.13-1865548, ONT). This workflow analyzed reads within 4649–5538 bp length range as defined in filtering condition (Supplementary Table S5). Based on WIMP taxonomic analysis, we calculated relative abundances (%) of the identified taxa by comparing the number of reads assigned to a particular taxon and the total number of reads obtained for the sample (Supplementary Table S5, Supplementary Fig. S2). Reads identified as *Homo sapiens* were filtered out from fastq files. The sequences of the final reads were deposited into the public NCBI SRA database and have the following accession number: PRJNA1081186.

### Supplementary Information

Supplementary Information.

## Data Availability

The Oxford Nanopore sequencing data included and discussed in the manuscript are deposited into the NCBI SRA database as a BioProject with accession number PRJNA1081186.

## Code availability

The code used for sequence scraping from online databases and nucleotide frequency calculation of aligned sequences is available as Custom code.txt file in Supplementary Material.
