# Supplementary material for: Harnessing the intragenomic variability of rRNA operons to improve differentiation of Vibrio species
Source: Sci Rep. 2024 Apr 30;14:9908. doi: 10.1038/s41598-024-60505-9 (PMC11061105; doi:10.1038/s41598-024-60505-9)
Supplement: Supplementary file 1 — Supplementary Information. [file 41598_2024_60505_MOESM1_ESM.pdf]

SUPPLEMENTARY MATERIAL

Supplementary Table S1. Summary of highly conserved and variable positions within 16S and 23S rRNA sequences of custom and SILVA databases. Highly conserved positions are defined as positions with the highest frequency value, x (i.e., x=1) and variable positions as the ten percent of positions with the lowest frequency values (i.e., x < 0.768 for 16S rRNA, x < 0.893 for representative *Vibrio* 23S rRNA and x < 0.747 for SILVA 23S rRNA sequences).

		Custom database		SILVA database
		**16S rRNA**	**23S rRNA**	**23S rRNA**
Highly conserved positions	Number (nts)	1210	2290	1743
	Percentage (%)	74.60	77.47	57.43
Variable positions	Number (nts)	162	295	303
	Percentage (%)	9.99	9.98	9.98
Total length with gaps (nts)		1622	2956	3035

Supplementary Table S2. Experimentally tested and putative oligonucleotides used as forward 16S rRNA gene-specific primers. Letters in bold represent the region covered by all primers; letters in red indicate degenerate nucleotides (R represents A or G; Y represents C or T; M represents A or C); the A in blue indicates a nucleotide conserved within *Vibrio* species.

Name (reference)	Sequence (5'-3')	Comments	References
27F (ref. 1)	___AGA**GTTTGATCMTGGC**TCAG	Proposed as a universal forward primer in 1991 (ref.1)	1–8
pA (ref. 10)	___AGA**GTTTGATCCTGGC**TCAG		2,3,9–13
8f (ref.14)	___AGA**GTTTGATYMTGGC**TCAG		3,14,15
A17 (ref. 16)	______**GTTTGATCCTGGC**TCAG		16
EB (ref. 17)	____GA**GTTTGATCCTGGC**TCAG		3,17
P0 (ref. 6)	____GA**GTTTGATCMTGGC**TCAG		3,6,18
GM3F (ref. 19)	___AGA**GTTTGATCMTGGC**_____	Reported as the most frequently used primer (see ref. 3)	3,8,19
S-DBact-0008-cS-20 (ref. 3)	___AG**RGTTYGATYMTGGC**TCAG	Provides the best coverage according to ref. 8	3,8
bio-pBR5'.SEF (ref. 20)	GAAGA**GTTTGATCATGGC**TCAG		3,20
9bF (ref. 21)	____G**RGTTTGATCCTGGC**TCAG		3,21
c27F	GAAG**RGTTYGATYMTGGC**TCAG	Consensus of bacterial primer sequences	This study
v27F	GAAGA**GTTTGATCATGG**CTCAG	*Vibrio*-specific consensus sequence	This study

Supplementary Table S3. Comparison of *Vibrio* 23S rRNA consensus sequences from custom and SILVA LSU Ref NR data repositories and selection of suitable 23S rRNA reverse *Vibrio*-specific primer. Letters in bold represent conserved nucleotides according to the database used; the letters in red indicate degenerate nucleotides (R can be A or G; Y can be C or T; H can be A, T or C; D can be G, A or T); the letters in blue indicate the positions of occasional indels. The 23S_rev_V primer sequence is the reverse complement of the unified consensus sequence.

**Conserved region coordinates (sense strand, without gaps)**	**Database**	***Vibrio* Consensus Sequence**	**Number of sequences with 100% match**	**Unified consensus sequence used for primer design**	**Primer name**	**Primer sequence**
2864-2889	Custom	5'-**YACTAATYGCCCRTGAGRCTTAACCA**-3'	421 out of 421	5'-**YACTAATYRCCCRTGAGDYYTA**-3'	23S_rev_V	5'-TARRHCTCAYGGGYRATTAGTR- 3'
	SILVA	5'-**TACTAA**T**TRCCCGTGA**G**DYYTA**A-**C**CA-3'	1903 out of 2072			

Supplementary Table S4. Non-*Vibrio* Vibrionaceae genomes yielding product of amplification with *Vibrio*-specific 27F (1) and 23S_rev_V primers.

Strain name	Genome source	Genome Accession number
Aliivibrio fischeri strain FQ-A001	NCBI	NZ_SJSX01000002
Aliivibrio salmonicida LFI1238	NCBI	NC_011312
Enterovibrio norvegicus isolate Alg239-V16	NCBI	NZ_UNRK01000018
Grimontia hollisae strain FDAARGOS_111	NCBI	NZ_CP014056
Photobacterium damselae strain 727-82	NCBI	NZ_JACGMH010000002

Supplementary Table S5. Summary of WIMP workflow results including quality control of reads and taxonomic assignment of classified reads. Reads assigned to humans have been excluded from read counts.

Quality Control (QC)						Taxonomic assignment of classified reads				
Total reads	Filtering conditions	Length failed qc filter (reads)	Workflow successful			Overview		Vibrionaceae reads		
	Length range (bp)		Total reads	Unclassified reads	Classified reads	Non-Vibrionaceae reads	Vibrionaceae reads	*Vibrio* read	Non-*Vibrio* Vibrionaceae read	Unclassified Vibrionaceae read
105,230	4649-5538	22,022	83,208	1	83,207	81,401 (97.83 %)	1,806 (2.17 %)	1,517 (84 %)	283 (15.67 %)	6 (0.33 %)

Supplementary Table S6. Taxonomic assignment of 1517 *Vibrio* reads representing the 16S-ITS-23S regions and their truncated variants (1600 nt) representing 16S rRNA gene sequences. The assigned *Vibrio* species and number of reads corresponding to these species obtained by Fastq WIMP analysis are indicated.

**Taxonomic assignment of 16S-ITS-23S reads**		**Taxonomic assignment of 16S-ITS-23S reads trimmed to 1600 nt (16S rRNA gene)**	
Vibrio species names	Read number	*Vibrio* species names	Read number
Vibrio splendidus	186	*Vibrio splendidus*	119
Vibrio atlanticus	175	*Vibrio cyclitrophicus*	111
Vibrio cyclitrophicus	169	*Vibrio anguillarum*	74
Vibrio anguillarum	108	*Vibrio atlanticus*	72
Vibrio alginolyticus	96	*Vibrio* sp. Sal 10	67
Vibrio scophthalmi	64	*Vibrio chagasii*	46

Vibrio sp. Sal 10	63	*Vibrio scophthalmi*	39
Vibrio chagasii	33	*Vibrio alginolyticus*	34
Vibrio nigripulchritudo	21	*Vibrio taketomensis*	11
Vibrio kanaloae	18	*Vibrio kanaloae*	9
Vibrio tapetis subsp. Tapetis	17	*Vibrio nigripulchritudo*	9
Vibrio mediterranei	15	*Vibrio europaeus*	9
Vibrio europaeus	11	Vibrio breoganii	8
Vibrio metoecus	11	*Vibrio tapetis* subp. tapetis	8
Vibrio sp. THAF191d	10	*Vibrio* sp. THAF191d	7
Vibrio taketomensis	9	*Vibrio aquimaris*	6
Vibrio breoganii	8	*Vibrio campbellii*	5
Vibrio rumoiensis	9	*Vibrio cholerae*	5
Vibrio astriarenae	8	*Vibrio campbellii* ATCC BAA-1116	4
Vibrio qinghaiensis	7	*Vibrio cincinnatiensis*	4
Vibrio aquimaris	6	*Vibrio coralliilyticus*	4
Vibrio panuliri	5	*Vibrio panuliri*	4
Vibrio campbellii ATCC BAA-1116	5	*Vibrio rumoiensis*	4
Vibrio parahaemolyticus	5	*Vibrio parahaemolyticus*	3
Vibrio sp. ZWAL4003	4	*Vibrio parahaemolyticus* UCM-V493	3
Vibrio cholerae IEC224	5	*Vibrio astriarenae*	3
Vibrio zhugei	4	*Vibrio spartinae*	3
Vibrio jasicida 090810c	4	*Vibrio zhugei*	3
Vibrio tubiashii ATCC 19109	4	*Vibrio natriegens*	2
Vibrio cholerae	4	*Vibrio* sp. THAF191c	2
Vibrio sp. THAF191c	3	*Vibrio* sp. ZWAL4003	2
Vibrio coralliilyticus	3	*Vibrio alfacsensis*	2
Vibrio ponticus	3	*Vibrio mediterranei*	2
Vibrio spartinae	3	*Vibrio metschnikovii*	2
Vibrio campbellii	2	*Vibrio campbellii* CAIM 519 = NBRC 15631	1

Vibrio owensii	2	*Vibrio diabolicus*	1
Vibrio parahaemolyticus UCM-V493	2	*Vibrio jasicida* 090810c	1
Vibrio sp. SM1977	2	*Vibrio* sp. SM1977	1
Vibrio aphrogenes	2	*Vibrio aphrogenes*	1
Vibrio cincinnatiensis	2	*Vibrio casei*	1
Vibrio gazogenes	1	*Vibrio cholerae* M66-2	1
Vibrio tritonius	2	*Vibrio furnissii* NCTC 11218	1
Vibrio vulnificus	2	*Vibrio gazogenes*	1
Vibrio diabolicus	1	*Vibrio navarrensis*	1
Vibrio natriegens	1	*Vibrio ponticus*	1
Vibrio rotiferianus	1	*Vibrio tritonius*	1
Vibrio sp. THAF190c	1	*Vibrio vulnificus*	1
Vibrio alfacsensis	1		
Vibrio cholerae M66-2	1		
Vibrio coralliilyticus OCN008	1		
Vibrio fluvialis	1		
Vibrio furnissii	1		
Vibrio metschnikovii	1		

Supplementary Table S7. List of all 16S and 23S rRNA gene pairs from representative *Vibrio* genomes used in the study.

#	Strain name	Genome source	Genome Accession number	16S rRNA gene Accession number	23S rRNA gene Accession number	Operon name
1	*Vibrio aestuarianus* subsp. francensis strain 02/041	NCBI	NZ_JAAZTV010000001.1	57793251	57793256	A
				57792612	57792610	B
				57790821	57790818	C
				57792957	57792954	D
				57793172	57793168	E
				57793106	57793104	F
2	*Vibrio alfacsensis* CAIM 1831^{T}	NCBI	CP032093.1	2860786713	2860786719	A
				2860786757	2860786760	B
				2860786820	2860786821	C
				2860786862	2860786866	D
				2860786868	2860786871	E
				2860787043	2860787044	F
				2860787046	2860787049	G
				2860787159	2860787160	H
				2860789235	2860789232	I
				2860789750	2860789746	J
			CP032094.1	2860789992	2860789998	K
3	*Vibrio alginolyticus* strain FDAARGOS_97	NCBI	NZ_LOSN02000001.1	61531378	61531381	A
				61530465	61530463	B

				61530641	61530640	C
				61530683	61530682	D
				61530882	61530886	E
				61530461	61530458	F
				61530638	61530633	G
				61530680	61530677	H
				61530368	61530365	I
				61530800	61530796	J
				61530759	61530756	K
			NZ_LOSN02000002.1	61534075	61534071	L
4	*Vibrio anguillarum* sv. 01 NB10	IMG	2645727930	2647597122	2647597124	A
				2647597203	2647597205	B
				2647597207	2647597212	C
				2647597275	2647597278	D
				2647597363	2647597366	E
				2647597778	2647597780	F
				2647599498	2647599495	G
				2647599973	2647599969	H
5	*Vibrio atlanticus* LGP32	NCBI	NC_011744.2	643499270	643499264	A
			NC_011753.2	643519804	643519809	B
				643520047	643520048	C
				643520295	643520297	D
				643522412	643522411	E
				643522673	643522670	F
				643522736	643522735	G
				643522778	643522774	H
6	*Vibrio azureus* LC2-005	NCBI	CP018616.1	2807429804	2807429808	A
				2807429810	2807429814	B
				2807429847	2807429851	C

				2807429900	2807429902	D
				2807429938	2807429939	E
				2807430096	2807430101	F
				2807430184	2807430188	G
				2807432045	2807432042	H
				2807432482	2807432478	I
			CP018617.1	2807433984	2807433979	J
7	*Vibrio breoganii* FF50	NCBI	CP016177.1	2813982968	2813982972	A
				2813983005	2813983007	B
				2813983048	2813983050	C
				2813983052	2813983055	D
				2813983210	2813983211	E
				2813983213	2813983216	F
				2813983218	2813983222	G
				2813985089	2813985084	H
				2813985528	2813985525	I
8	*Vibrio campbellii* strain BoB-53	NCBI	NZ_CP026321.1	67375864	67375868	A
				67375749	67375753	B
				67376182	67376185	C
				67376086	67376089	D
				67375910	67375913	E
				67375790	67375793	F
				67375860	67375862	G
				67376083	67376084	H
				67375907	67375908	I
				67378363	67378360	J
				67378854	67378849	K
			NZ_CP026322.1	67380651	67380656	L
9	*Vibrio casei* DSM 22364	NCBI	AP018680.1	2860767661	2860767666	A

				2860768049	2860768052	B
				2860768054	2860768058	C
				2860769733	2860769729	D
				2860770173	2860770170	E
				2860770207	2860770205	F
				2860770222	2860770220	G
			AP018681.1	2860770443	2860770445	H
10	*Vibrio chagasii* ECSMB14107	NCBI	CP034970.1	2845268209	2845268213	A
				2845268260	2845268261	B
				2845268263	2845268266	C
				2845268345	2845268346	D
				2845268348	2845268351	E
				2845268353	2845268357	F
				2845268637	2845268639	G
				2845268641	2845268644	H
				2845271059	2845271057	I
				2845271345	2845271342	J
				2845271349	2845271347	K
				2845271631	2845271626	L
			CP034971.1	2845272291	2845272295	M
11	*Vibrio cholera* V060002	IMG	AP018677.1	2912595183	2912595185	A
				2912595279	2912595281	B
				2912595283	2912595288	C
				2912595534	2912595537	D
				2912598235	2912598233	E
				2912598575	2912598571	F
				2912598580	2912598577	G
				2912598664	2912598661	H
				2912598831	2912598827	I

12	*Vibrio coralliilyticus* strain S2052	NCBI	NZ_CP063051.1	66920587	66920584	A
				66920591	66920589	B
				66920655	66920654	C
				66918153	66918156	D
				66918025	66918028	E
				66920839	66920843	F
				66920582	66920578	G
				66920652	66920649	H
				66920760	66920755	I
13	*Vibrio crassostreae* 9CS106	IMG	CP016228.1	2912636054	2912636051	A
				2912636057	2912636056	B
				2912636061	2912636059	C
				2912636328	2912636323	D
				2912636407	2912636411	E
				2912636456	2912636457	F
				2912636459	2912636462	G
				2912636523	2912636524	H
				2912636526	2912636529	I
				2912636531	2912636535	J
				2912636794	2912636796	K
				2912636798	2912636801	L
				2912638925	2912638923	M
				2912639162	2912639159	N
				2912639165	2912639164	O
			CP016229.1	2912640320	2912640314	P
14	*Vibrio cyclitrophicus* strain ECSMB14105	NCBI	NZ_CP039700.1	50228582	50228583	A
				50228646	50228647	B
				50231152	50231151	C
				50228904	50228906	D

				50231156	50231154	E
				50228585	50228588	F
				50228649	50228652	G
				50228908	50228911	H
				50228541	50228545	I
				50228654	50228658	J
				50231149	50231146	K
				50231443	50231438	L
				50230917	50230915	M
			NZ_CP039701.1	50231803	50231797	N
15	*Vibrio diabolicus* FDAARGOS_96	NCBI	CP014095.1	2807697308	2807697304	A
			CP014094.1	2807698598	2807698603	B
				2807698643	2807698646	C
				2807698726	2807698727	D
				2807698729	2807698733	E
				2807698771	2807698774	F
				2807698776	2807698779	G
				2807698951	2807698952	H
				2807698954	2807698957	I
				2807699053	2807699056	J
				2807701106	2807701103	K
				2807701638	2807701634	L
16	*Vibrio fluvialis* strain ATCC 33809^T	NCBI	NZ_CP014035.2	29385641	29387470	A
				29386129	29386932	B
				29387232	29385226	C
				29386671	29386169	D
				29385999	29387272	E
				29386091	29386014	F
				29385117	29385899	G

				29386381	29385987	H
				29387227	29386640	I
				29385855	29385605	J
17	*Vibrio furnissii* strain FDAARGOS_777	NCBI	NZ_CP040990.1	50534124	50534128	A
				50534367	50534370	B
				50533895	50533893	C
				50534363	50534365	D
				50534044	50534040	E
				50533891	50533886	F
				50534038	50534035	G
				50536452	50536450	H
				50533623	50533620	I
				50534276	50534279	J
18	*Vibrio gazogenes* ATCC 43942	IMG	CP018835.1	2775355655	2775355657	A
				2775356043	2775356047	B
				2775356277	2775356280	C
				2775358373	2775358369	D
				2775358378	2775358375	E
				2775358542	2775358540	F
				2775358547	2775358544	G
				2775358742	2775358738	H
19	*Vibrio harveyi* strain ATCC 33843 (392 [MAV])	NCBI	NZ_CP009467.1	57819754	57819759	A
				57820236	57820239	B
				57822628	57822627	C
				57819532	57819531	D
				57819577	57819575	E
				57822625	57822622	F
				57819573	57819572	G
				57822534	57822531	H

				57819636	57819633	I
				57819675	57819671	J
				57819529	57822818	K
			NZ_CP009468.1	57823910	57823915	L
20	*Vibrio jasicida* 090810c	NCBI	NZ_CP025792.1	48229652	48229653	A
				48229613	48229614	B
				48229834	48229836	C
				48229838	48229841	D
				48229553	48229556	E
				48229661	48229664	F
				48229929	48229932	G
				48229655	48229659	H
				48229513	48229518	I
				48228934	48228931	J
				48229416	48229412	K
			NZ_CP025793.1	48232791	48232786	L
21	*Vibrio kanaloae* strain R17	NCBI	NZ_CP065150.1	61412707	61412711	A
				61412818	61412822	B
				61413071	61413074	C
				61412750	61412753	D
				61413067	61413069	E
				61412380	61412378	F
				61412383	61412382	G
				61412747	61412748	H
				61412815	61412816	I
				61412812	61412813	J
				61412376	61412373	K
				61412107	61412104	L
				61412629	61412624	M

			NZ_CP065151.1	61414618	61414613	N
22	*Vibrio mediterranei* QT6D1	IMG	NZ_CP018308.1	2775372373	2775372377	A
				2775372494	2775372496	B
				2775372498	2775372502	C
				2775372504	2775372507	D
				2775375151	2775375148	E
				2775375362	2775375359	F
				2775375366	2775375364	G
				2775375370	2775375368	H
				2775375416	2775375414	I
				2775375657	2775375654	J
				2775375697	2775375692	K
23	*Vibrio metschnikovii* strain 9502-00	NCBI	NZ_CP046793.1	58785665	58785669	A
				58785205	58785208	B
				58785460	58785463	C
				58785018	58785013	D
				58785210	58785212	E
				58785092	61971052	F
				58784797	58784793	G
				58784714	58784711	H
				58785011	58785009	I
24	*Vibrio mimicus* MB451	NCBI	NZ_ADAF01000001.1	57836834	57836839	A
				57836603	57836607	B
				57837154	57837157	C
				57836753	57836755	D
				57838771	57838769	E
				57836517	57836515	F
25	*Vibrio natriegens* CCUG 16374	NCBI	CP016351.1	2813784218	2813784222	A
				2813784827	2813784830	B

				2813786912	2813786909	C
				2813787004	2813787001	D
				2813787007	2813787006	E
				2813787180	2813787177	F
				2813787183	2813787182	G
				2813787224	2813787220	H
				2813787228	2813787226	I
				2813787284	2813787281	J
				2813787328	2813787323	K
			CP016352.1	2813787801	2813787796	L
26	*Vibrio navarrensis* strain 0053-83	NCBI	NZ_CP051121.1	59229767	59229772	A
				59229904	59229908	B
				59229910	59229913	C
				59229808	59229811	D
				59229416	59229413	E
				59229901	59229902	F
				59230205	59230206	G
				59229411	59229407	H
				59229679	59229676	I
				59229210	59229209	J
			NZ_CP051120.1	59228348	59228343	K
27	*Vibrio neocaledonicus* CGJ02-2	NCBI	CP032213.1	2875330897	2875330901	A
				2875331399	2875331402	B
				2875333670	2875333667	C
				2875333764	2875333761	D
				2875333768	2875333766	E
				2875333943	2875333938	F
				2875333946	2875333945	G
				2875333988	2875333985	H

				2875333991	2875333990	I
				2875334051	2875334048	J
				2875334094	2875334090	K
			CP032214.1	2875335178	2875335174	L
28	*Vibrio nigripulchritudo* SFn1	IMG	FO203526.1	2563697469	2563697471	A
				2563697819	2563697822	B
				2563700360	2563700357	C
				2563700654	2563700649	D
				2563700661	2563700656	E
				2563700817	2563700815	F
				2563701081	2563701077	G
				2563701127	2563701124	H
29	*Vibrio owensii* strain XSBZ03	NCBI	NZ_CP019959.1	47100157	47100160	A
				47099980	47099983	B
				47100249	47100252	C
				47099935	47099939	D
				47099831	47099835	E
				47099931	47099933	F
				47099977	47099978	G
				47100154	47100155	H
				47099267	47099264	I
				47099751	47099746	J
				47099871	47099874	K
			NZ_CP019960.1	47101438	47099746	L
30	*Vibrio panuliri* JCM 19500^T	NCBI	AP019654.1	2896912519	2896912524	A
				2896912783	2896912784	B
				2896912786	2896912788	C
				2896912790	2896912793	D
				2896914904	2896914901	E

				2896915053	2896915050	F
				2896915331	2896915328	G
				2896915335	2896915334	H
				2896915398	2896915393	I
				2896915403	2896915400	J
				2896915508	2896915503	K
31	*Vibrio parahaemolyticus* RIMD 2210633	NCBI	NC_004603.1	1187490	1187494	A
				1188031	1188034	B
				1190520	1190519	C
				1190464	1190463	D
				1190517	1190516	E
				1190461	1190456	F
				1190269	1190267	G
				1190585	1190582	H
				1190174	1190171	I
				1190629	1190625	J
			NC_004605.1	1190822	1190827	K
32	*Vibrio rotiferianus* strain B64D1	NCBI	NZ_CP018312.1	47658382	47658383	A
				47658430	47658431	B
				47655559	47655560	C
				47655562	47655564	D
				47658322	47658325	E
				47658391	47658394	F
				47655655	47655658	G
				47655391	47655394	H
				47658385	47658389	I
				47658283	47658287	J
				47658188	47658183	K
				47657708	47657705	L

			NZ_CP018311.1	47654641	47654646	M
33	*Vibrio rumoiensis* FERM P-14531^T	NCBI	AP018685.1	2877629614	2877629618	A
				2877629632	2877629635	B
				2877629667	2877629670	C
				2877631576	2877631571	D
				2877631761	2877631759	E
				2877631765	2877631763	F
				2877631769	2877631767	G
				2877632106	2877632105	H
34	*Vibrio scophthalmi* VS-12	NCBI	CP016307.1	2721583315	2721583319	A
				2721583570	2721583571	B
				2721583573	2721583575	C
				2721583577	2721583578	D
				2721583844	2721583847	E
				2721585760	2721585757	F
				2721586075	2721586072	G
				2721586081	2721586077	H
				2721586084	2721586083	I
				2721586148	2721586145	J
				2721586153	2721586150	K
				2721586271	2721586266	L
				2721586274	2721586273	M
35	*Vibrio splendidus* BST398	NCBI	CP031055.1	2896917036	2896917037	A
				2896917039	2896917041	B
				2896917043	2896917046	C
				2896917284	2896917286	D
				2896919513	2896919510	E
				2896919516	2896919515	F
				2896919772	2896919768	G

				2896919777	2896919774	H
				2896919780	2896919779	I
				2896919783	2896919782	J
				2896919846	2896919843	K
				2896919849	2896919848	L
				2896919893	2896919889	M
				2896919974	2896919979	N
			CP031056.1	2896921802	2896921796	O
36	*Vibrio tapetis tapetis* CECT 4600^T	NCBI	LT960611.1	2850901076	2850901079	A
				2850903549	2850903546	B
				2850904268	2850904263	C
				2850904356	2850904360	D
				2850904384	2850904386	E
37	*Vibrio tritonius* AM2^T	NCBI	AP014635.1	2672406127	2672406131	A
				2672406320	2672406322	B
				2672406324	2672406327	C
				2672406413	2672406415	D
				2672406417	2672406418	E
				2672408617	2672408615	F
				2672408810	2672408807	G
				2672409054	2672409051	H
				2672409060	2672409056	I
				2672409175	2672409172	J
				2672409183	2672409178	K
38	*Vibrio tubiashii* ATCC 19109^T	NCBI	CP009354.1	2629905388	2629905392	A
				2629905632	2629905634	B
				2629905636	2629905639	C
				2629905907	2629905910	D
				2629907927	2629907924	E

				2629908205	2629908202	F
				2629908209	2629908207	G
				2629908272	2629908268	H
				2629908277	2629908274	I
				2629908377	2629908372	J
39	*Vibrio vulnificus* strain FORC_037	NCBI	NZ_CP016321.1	66963556	66963558	A
				66963982	66963984	B
				66966153	66966150	C
				66963514	66963516	D
				66966376	66966372	E
				66963551	66963554	F
				66966148	66966143	G
				66966370	66966367	H
			NZ_CP016322.1	66966811	66966816	I
			NZ_CP016321.1	66966502	66966498	J
				66966072	66966069	K
40	*Vibrio zhugei* HBUAS61001^T	NCBI	CP033078.1	2870259485	2870259489	A
				2870259491	2870259494	B
				2870259651	2870259654	C
				2870259735	2870259737	D
				2870259739	2870259742	E
				2870261587	2870261585	F
				2870261804	2870261801	G
				2870262055	2870262051	H
				2870262209	2870262204	I
O1	*Escherichia coli* O157:H7 strain MB9-1	NCBI	NZ_CP040107.1	CP040107 Reg.: 4536303..4537840	CP040107 Reg.: 4532952..4535853	
O2	*Salmonella bongori* N268-08	NCBI	NC_021870.1	CP006608 Reg.: 3943428..3944967	CP006608 Reg.: 3945313..3948215	

Supplementary Table S8. Criteria used for the taxonomic assignment of *Vibrio* species that during phylogenetic reconstruction of 16S and 23S rRNA gene-based trees produced insufficiently supported monophyletic clades, polyphyletic clades or other types of ambiguities. The species taxonomic assignment is supported by literature data and NCBI criterion (no asterisk), satisfies only the NCBI criterion (*) or does not satisfy any of the criteria (**). The assembly quality assessment in NCBI is explained at: https://www.ncbi.nlm.nih.gov/assembly/help/.

Representative *Vibrio* species	Strain name	Genome Source	Reference		Assembly Quality Assessment (AQA) according to the NCBI criteria				
			Sufficient experimental evidence	Number	Best-matching type strain assembly	ANI	Query Coverage	Subject coverage	Taxonomy status
V. aestuarianus	subsp. Francensis 02/041T VIBAE_A.3	NCBI	Yes	22	*Vibrio coralliilyticus* ATCC BAA-450	96.88	89.94	86.08	OK, species match
V. alfacsensis	CAIM 1831^{T}	NCBI (ref IMG/M)	Yes	23	*Vibrio alfacsensis*	96.71	80.14	85.9	OK, species match
*V. alginolyticus**	FDAARGO S_97	NCBI	No	24	*Vibrio alginolyticus* NBRC 15630	100	97.5	99.97	OK, species match
V. atlanticus,1*	LGP32	NCBI (ref IMG/M)	No	25	*Vibrio atlanticus* CECT 7223	96.29	85.21	85	OK, species match
*V. azureus**	LC2-005	NCBI (ref IMG/M)	No	26	*Vibrio azureus* NBRC 104587	100	97.56	99.98	OK, species match
*V. breoganii***	FF50	NCBI (ref IMG/M)	No	_	*Vibrio superstes*	92.63	73.6	70.37	Inconclusive, below-threshold match
V. campbellii	BoB-53	NCBI	Yes	27	*Vibrio campbellii* CAIM 519	96.54	81.05	86.63	OK, species match

V. chagasii	ECSMB14 107	NCBI (ref IMG/M)	Yes	28	*Vibrio chagasii*	93.93	76.97	79.64	Inconclusive, below-threshold match
V. cholerae	V060002	NCBI (ref IMG/M)	Yes	29	*Vibrio cholerae* ATCC 14035	99.37	94.77	95.6	OK, species match
*V. coralliilyticus**	S2052	NCBI	No	30	*Vibrio coralliilyticus* ATCC BAA-450	96.88	89.94	89.94	OK, species match
*V. crassostreae***	9CS106	NCBI (ref IMG/M)	No	31	*Vibrio coralliirubri*	94.71	79.09	77.1	Inconclusive, below-threshold match
*V. cyclitrophicus***	ECSMB14 105	NCBI	No	32	*Vibrio echinoideorum*	89.11	82.4	74.33	Inconclusive, below-threshold match
*V. diabolicus***	FDAARGO S_96	NCBI (ref IMG/M)	No	_	*Vibrio chemaguriensis*	97.96	89.36	92.35	Inconclusive, below-threshold match
V. harveyi	ATCC 33843	NCBI	Yes	33,34	*Vibrio harveyi* NBRC 15634	98.8	90.52	95.35	OK, species match
V. jasicida	090810c	NCBI	Yes	35	*Vibrio jasicida*		94.78	95.5	OK, species match
*V. kanaloae**	R17	NCBI	No	_	*Vibrio kanaloae*	98.3	90.45	92.2	OK, species match
V. mediterranei	QT6D1	NCBI (ref IMG/M)	Yes	36	*Vibrio mediterranei* NBRC 15635	97.53	86.88	89.7	OK, species match
V. natriegens	CCUG 16374	NCBI (ref IMG/M)	Yes	37	*Vibrio natriegens* NBRC 15636	98.16	84.67	90.17	OK, species match
*V. navarrensis**	0053-83	NCBI	No	_	*Vibrio navarrensis*	97.28	86.42	83.46	OK, species match

*V. neocaledonicus***, *2*	CGJ02-2	NCBI (ref IMG/M)	No	38	*Vibrio alginolyticus*	98.51	89.96	93.45	Inconclusive, below-threshold match
V. nigripulchritudo	SFn1	NCBI (ref from IMG/M)	Yes	39	*Vibrio nigripulchritudo* ATCC 27043	97.09	89.97	93.04	OK, species match
*V. owensii**	XSBZ03	NCBI	No	_	*Vibrio owensii* CAIM 1854	96.89	86.38	80.18	OK, species match
*V. panuliri**	JCM 19500^T	NCBI	No	_	*Vibrio panuliri*	99.99	99.03	99.96	OK, species match
V. rotiferianus	B64D1	NCBI	Yes	36	*Vibrio rotiferianus* CAIM 577	97.42	87.8	86.32	OK, species match
*V. splendidus**	BST398	NCBI (ref IMG/M)	No	_	*Vibrio splendidus*	94.67	84.07	80.84	OK, species match
V. tapetis	CECT4600^T	NCBI (ref IMG/M)	Yes	40	*Vibrio splendidus*	83.89	6.04	6.03	OK, low-coverage
*V. tubiashii**	ATCC 19109^T	NCBI (ref IMG/M)	No	41	*Vibrio tubiashii* ATCC 19109	100	98.6	99.92	OK, species match
*V. vulnificus**	FORC_037	NCBI	No	_	*Vibrio vulnificus*	97.32	87.09	89.34	OK, species match

1 The strain LGP32 is originally described as *V. splendidus* but according to the NCBI database is annotated as *V. atlanticus* (https://www.ncbi.nlm.nih.gov/nuccore/NC_011744).

2 The CGJ02-2 strain was previously classified *as V. neocaledonicus* but finally assigned to *V. alginolyticus*[38]. Still the NCBI entry for CGJ02-2 strain is annotated as *V. neocaledonicus* (https://www.ncbi.nlm.nih.gov/nuccore/CP032213.1/).

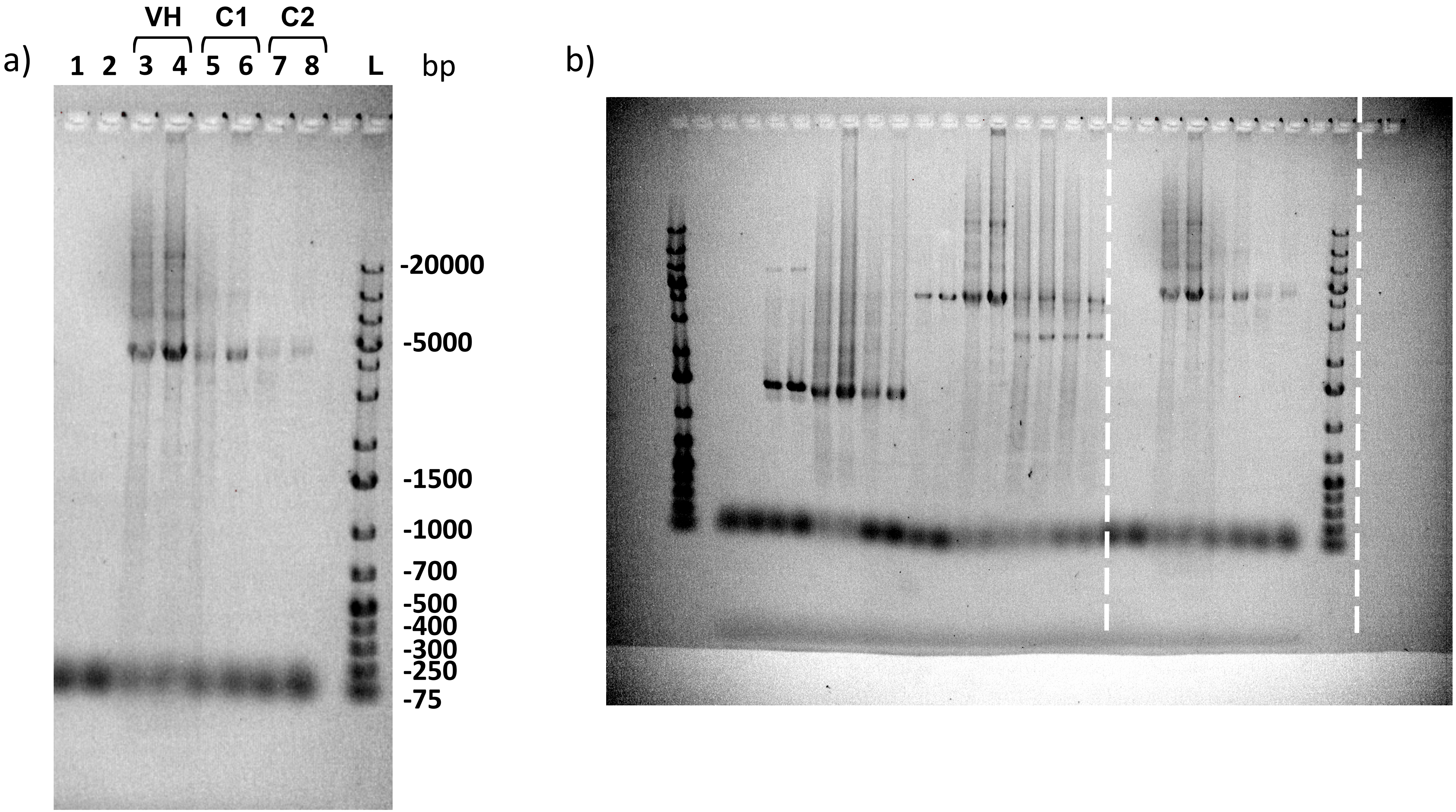

Supplementary Figure S1. Agarose gel electrophoresis of DNA fragments generated by PCR amplification of metagenomic DNA using 27F[1] and 23S_rev_V primers and high-fidelity Platinum SuperFi II DNA polymerase. ***(a)*** Lanes with odd and even numbering correspond to PCR products before and after their deproteinization, respectively. Deproteinization was carried out with undiluted (C1) and diluted (1:10, C2) products of amplification. Lanes 1 and 2, negative controls, i.e., PCR amplifications carried out in the absence of template DNA. Lanes 3 and 4, PCR products obtained with V. harveyi genomic DNA (VH, positive control). Lane L, 1 kb ladder.
(b) Original gel image. The cropped area (shown in panel ***a***) is flanked by dashed lines.

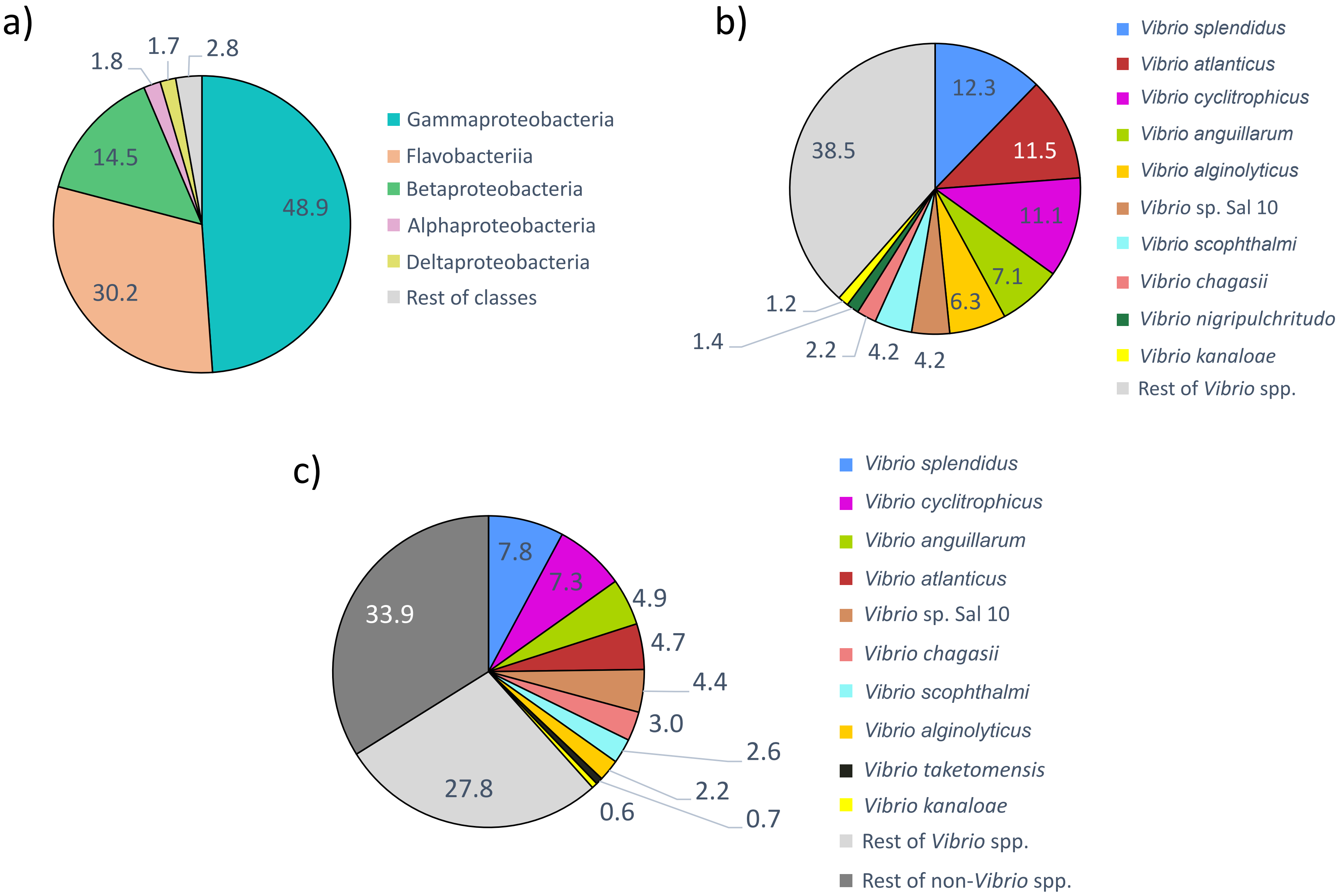

Supplementary Figure S2. Taxonomic classification of MinION 16S-ITS-23S reads (a, b) and their truncated variants containing 16S sequences (c) obtained with the ES_Ple_Mar environmental sample. The taxonomic assignment of 83,207 reads by WIMP (Oxford Nanopore) reveals the relative abundance (%) of different classes of bacteria shown as diagram sectors in panel ***a***. The diagram of panel ***b*** provides further details regarding the presence of individual *Vibrio* species based on 1,517 *Vibrio* reads. The subsequent trimming of these reads from 3’ end to obtain 1600 bp fragments (16S rRNA) followed by their annotation by WIMP considerably reduces the total number of *Vibrio* reads and species diversity in panel ***c***.

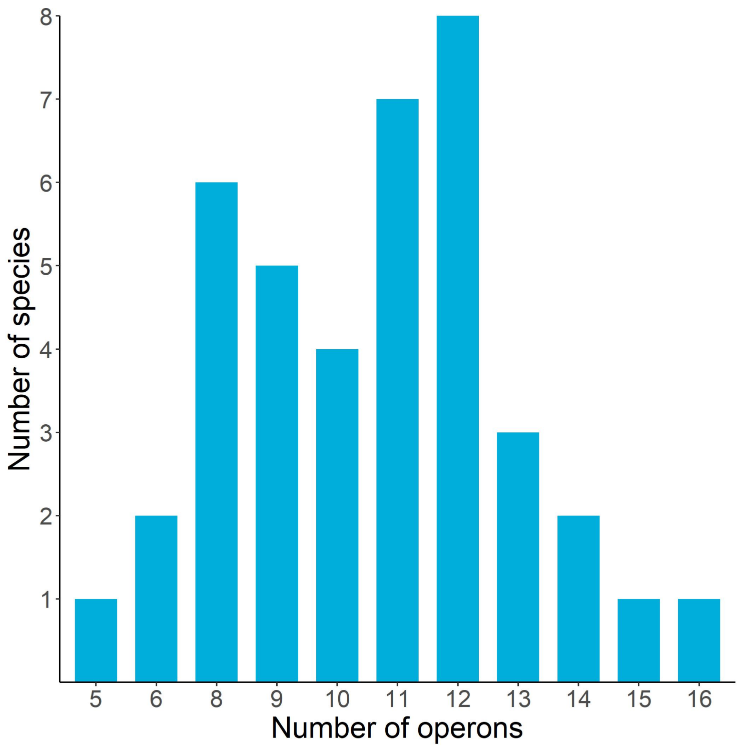

Supplementary Figure S3. Distribution of ribosomal operon copy number among representative *Vibrio* species.

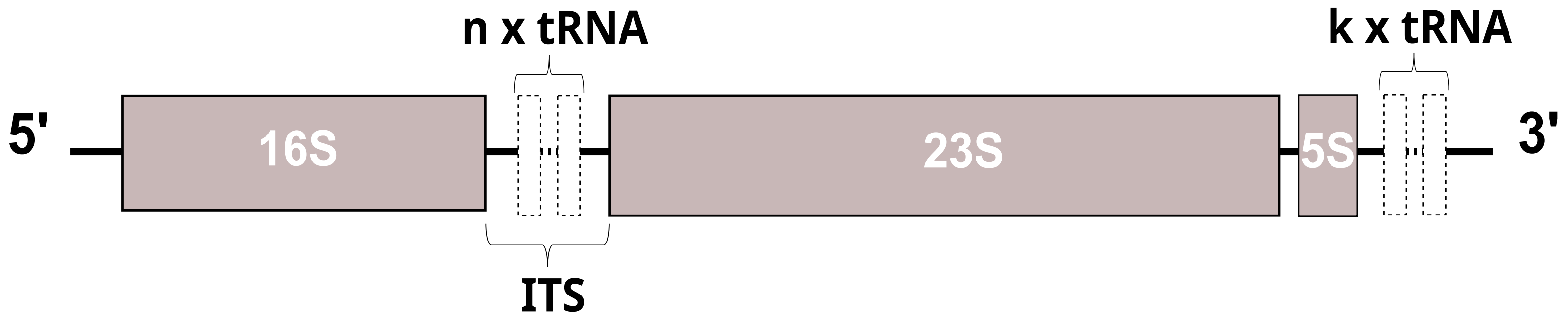

Supplementary Figure S4. Ribosomal operon structure revealed by analysis of 40 representative *Vibrio* species. 16S and 23S rRNA genes are separated by an internal transcribed spacer (ITS) containing one to five ($n \leq 5$) copies of tRNA genes, whereas other tRNA gene copies ($k \leq 2$) can be found after 5S rRNA gene.

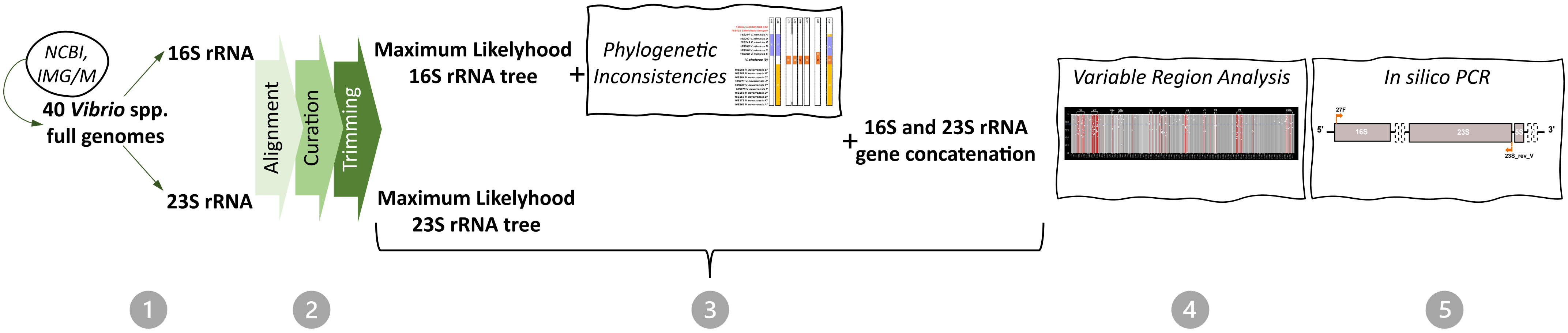

Supplementary Figure S5. Pipeline of the methodology used in this study. (1) 16S rRNA and 23S rRNA gene sequence retrieval from 40 representative *Vibrio* genomes available in NCBI and IMG/M databases**.** (2) Sequence alignment, curation and trimming for (3) Maximum likelihood phylogenetic tree construction and posterior analysis of phylogenetic ambiguities encountered in the 16S rRNA tree. Concatenation of 16S and 23S rRNA gene sequences from the same operon and tree construction. (4) Identification of conserved and variable regions. (5) Selection of primer target sites and *in silico* PCR for amplification of *Vibrio* 16S-23S regions.

Supplementary References

1. Lane, D.J. 16S/23S rRNA Sequencing in Nucleic Acid Techniques in *Bacterial Systematics* (eds. Stackebrandt, E. & Goodfellow, M.) 115-175 (John Wiley and Sons, 1991).
2. Fu, K. et al. An Innovative Method for Rapid Identification and Detection of Vibrio alginolyticus in Different Infection Models. *Front. Microbiol.* **7**, (2016).
3. Klindworth, A. et al. Evaluation of general 16S ribosomal RNA gene PCR primers for classical and next-generation sequencing-based diversity studies. *Nucleic Acids Research* **41**, e1–e1 (2013).
4. Osman, E. et al. Molecular approaches for detection and quantification of Vibrio scophthalmi based on recA. *Journal of Fish Diseases* **45**, 373–378 (2022).
5. Suzuki, M. T. & Giovannoni, S. J. Bias caused by template annealing in the amplification of mixtures of 16S rRNA genes by PCR. *Appl. Environ. Microbiol.* **62**, 625–630 (1996).
6. Frank, J. A. et al. Critical Evaluation of Two Primers Commonly Used for Amplification of Bacterial 16S rRNA Genes. *Appl. Environ. Microbiol.* **74**, 2461–2470 (2008).
7. Yen, P. T. H., Linh, N. Q. & Tram, N. D. Q. The identification and determination of toxin genes of Vibrio strains causing hemorrhagic disease on red drum (Sciaenops ocellatus) using PCR. *AMB Expr.* **11**, 8 (2021).

8. Karst, S. M. et al. Retrieval of a million high-quality, full-length microbial 16S and 18S rRNA gene sequences without primer bias. *Nat. Biotechnol.* **36**, 190–195 (2018).

9. Hayashi, H., Sakamoto, M. & Benno, Y. Phylogenetic Analysis of the Human Gut Microbiota Using 16S rDNA Clone Libraries and Strictly Anaerobic Culture-Based Methods. *Microbiology and Immunology* **46**, 535–548 (2002).

10. Edwards, U., Rogall, T., Blöcker, H., Emde, M. & Böttger, E. Isolation and direct complete nucleotide determination of entire genes. Characterization of a gene coding for 16S ribosomal RNA. *Nucleic Acids Res.* **17**, 7843–53 (1989).

11. Hang, J. et al. 16S rRNA gene pyrosequencing of reference and clinical samples and investigation of the temperature stability of microbiome profiles. *Microbiome* **2**, 31 (2014).

12. Isenbarger, T. A., Finney, M., Ríos-Velázquez, C., Handelsman, J. & Ruvkun, G. Miniprimer PCR, a New Lens for Viewing the Microbial World. *Appl. Environ. Microbiol.* **74**, 840–849 (2008).

13. Weisburg, W. G., Barns, S. M., Pelletier, D. A. & Lane, D. J. 16S ribosomal DNA amplification for phylogenetic study. *J. Bacteriol.* **173**, 697–703 (1991).

14. Kim, Y. M., Ahn, C. K., Woo, S. H., Jung, G. Y. & Park, J. M. Synergic degradation of phenanthrene by consortia of newly isolated bacterial strains. *Journal of Biotechnology* **144**, 293–298 (2009).

15. Juretschko, S. et al. Combined Molecular and Conventional Analyses of Nitrifying Bacterium Diversity in Activated Sludge: Nitrosococcus mobilis and Nitrospira -Like Bacteria as Dominant Populations. *Appl. Environ. Microbiol.* **64**, 3042–3051 (1998).

16. Kumar, P. S., Griffen, A. L., Moeschberger, M. L. & Leys, E. J. Identification of Candidate Periodontal Pathogens and Beneficial Species by Quantitative 16S Clonal Analysis. *J. Clin. Microbiol.* **43**, 3944–3955 (2005).

17. McInerney, J. O., Wilkinson, M., Patching, J. W., Embley, T. M. & Powell, R. Recovery and phylogenetic analysis of novel archaeal rRNA sequences from a deep-sea deposit feeder. *Appl. Environ. Microbiol.* **61**, 1646–1648 (1995).

18. Lee, T. K. et al. Discovery of commonly existing anode biofilm microbes in two different wastewater treatment MFCs using FLX Titanium pyrosequencing. *Appl. Microbiol. Biotechnol.* **87**, 2335–2343 (2010).

19. Muyzer, G., Teske, A., Wirsen, C. O. & Jannasch, H. W. Phylogenetic relationships of Thiomicrospira species and their identification in deep-sea hydrothermal vent samples by denaturing gradient gel electrophoresis of 16S rDNA fragments. *Arch. Microbiol.* **164**, 165–172 (1995).

20. Jonasson, J., Olofsson, M. & Monstein, H.-J. Classification, identification and subtyping of bacteria based on pyrosequencing and signature matching of 16S rDNA fragments. *APMIS* **110**, 263–272 (2002).

21. Huber, H. et al. A new phylum of Archaea represented by a nanosized hyperthermophilic symbiont. *Nature* **417**, 63–67 (2002).

22. Garcia, C. et al. Vibrio aestuarianus subsp. cardii subsp. nov., pathogenic to the edible cockles Cerastoderma edule in France, and establishment of Vibrio aestuarianus subsp. aestuarianus subsp. nov. and Vibrio aestuarianus subsp. francensis subsp. nov. *International Journal of Systematic and Evolutionary Microbiology* **71**, (2021).

23. Gomez-Gil, B. et al. Vibrio alfacsensis sp. nov., isolated from marine organisms. *International Journal of Systematic and Evolutionary Microbiology* **62**, 2955–2961 (2012).

24. Liu, X.-F., Cao, Y., Zhang, H.-L., Chen, Y.-J. & Hu, C.-J. Complete Genome Sequence of Vibrio alginolyticus ATCC 17749T. *Genome Announcements* **3**, e01500-14 (2015).
25. Le Roux, F. et al. Genome sequence of Vibrio splendidus : an abundant planctonic marine species with a large genotypic diversity. *Environmental Microbiology* **11**, 1959–1970 (2009).
26. Urbanczyk, H., Ogura, Y. & Hayashi, T. Taxonomic revision of Harveyi clade bacteria (family Vibrionaceae) based on analysis of whole genome sequences. *International Journal of Systematic and Evolutionary Microbiology* **63**, 2742–2751 (2013).
27. Colston, S. M. et al. Complete Genome Sequences of Two Bioluminescent Vibrio campbellii Strains Isolated from Biofouling Communities in the Bay of Bengal. *Genome Announc.* **6**, e00422-18 (2018).
28. Liang, X. et al. Complete genome of a marine bacterium Vibrio chagasii ECSMB14107 with the ability to infect mussels. *Marine Genomics* **48**, 100683 (2019).
29. Yamamoto, S. et al. Single Circular Chromosome Identified from the Genome Sequence of the Vibrio cholerae O1 bv. El Tor Ogawa Strain V060002. *Genome Announc.* **6**, e00564-18 (2018).
30. Machado, H., Sonnenschein, E. C., Melchiorsen, J. & Gram, L. Genome mining reveals unlocked bioactive potential of marine Gram-negative bacteria. *BMC Genomics* **16**, 158 (2015).
31. Cordero, O. X. et al. Ecological Populations of Bacteria Act as Socially Cohesive Units of Antibiotic Production and Resistance. *Science* **337**, 1228–1231 (2012).

32. Hehemann, J.-H. et al. Adaptive radiation by waves of gene transfer leads to fine-scale resource partitioning in marine microbes. *Nat. Commun.* **7**, 12860 (2016).

33. Wang, Z., Hervey, W. J., Kim, S., Lin, B. & Vora, G. J. Complete Genome Sequence of the Bioluminescent Marine Bacterium Vibrio harveyi ATCC 33843 (392 [MAV]). *Genome Announc.* **3**, e01493-14 (2015).

34. Reichelt, J. L. & Baumann, P. Taxonomy of the marine, luminous bacteria. *Archiv. Mikrobiol.* **94**, 283–330 (1973).

35. Ke, H.-M. et al. Tracing Genomic Divergence of Vibrio Bacteria in the Harveyi Clade. *J. Bacteriol.* **200**, e00001-18 (2018).

36. Lin, H., Yu, M., Wang, X. & Zhang, X.-H. Comparative genomic analysis reveals the evolution and environmental adaptation strategies of vibrios. *BMC Genomics* **19**, 135 (2018).

37. Weinstock, M. T., Hesek, E. D., Wilson, C. M. & Gibson, D. G. Vibrio natriegens as a fast-growing host for molecular biology. *Nat. Methods* **13**, 849–851 (2016).

38. Tan, L. et al. Complete Genome of Vibrio neocaledonicus CGJ02-2, An active Compounds Producing Bacterium Isolated from South China Sea. *Curr. Microbiol.* **77**, 2665–2673 (2020).

39. Goudenège, D. et al. Comparative genomics of pathogenic lineages of Vibrio nigripulchritudo identifies virulence-associated traits. *ISME J.* **7**, 1985–1996 (2013).

40. Dias, G. M. et al. Vibrio tapetis Displays an Original Type IV Secretion System in Strains Pathogenic for Bivalve Molluscs. *Front. Microbiol.* **9**, 227 (2018).

41. Richards, G. P., Needleman, D. S., Watson, M. A. & Bono, J. L. Complete Genome Sequence of the Larval Shellfish Pathogen Vibrio tubiashii Type Strain ATCC 19109. *Genome Announc.* **2**, e01252-14 (2014).
